# In Praise of Artifice Reloaded: Caution With Natural Image Databases in Modeling Vision

**DOI:** 10.3389/fnins.2019.00008

**Published:** 2019-02-18

**Authors:** Marina Martinez-Garcia, Marcelo Bertalmío, Jesús Malo

**Affiliations:** ^1^Image Processing Lab, Universitat de València Valencia, Spain; ^2^CSIC, Instituto de Neurociencias Alicante, Spain; ^3^Departamento de Tecnologías de la Información y las Comunicaciones, Universidad Pompeu Fabra Barcelona, Spain

**Keywords:** natural stimuli, artificial stimuli, subjective image quality databases, wavelet + divisive normalization, contrast masking

## Abstract

Subjective image quality databases are a major source of raw data on how the visual system works in *naturalistic environments*. These databases describe the sensitivity of many observers to a wide range of distortions of different nature and intensity seen on top of a variety of natural images. Data of this kind seems to open a number of possibilities for the vision scientist to check the models in realistic scenarios. However, while these natural databases are great benchmarks for models developed in some other way (e.g., by using the well-controlled *artificial stimuli* of traditional psychophysics), they should be carefully used when trying to fit vision models. Given the high dimensionality of the image space, it is very likely that some basic phenomena are under-represented in the database. Therefore, a model fitted on these large-scale natural databases will not reproduce these under-represented basic phenomena that could otherwise be easily illustrated with well selected artificial stimuli. In this work we study a specific example of the above statement. A standard cortical model using wavelets and divisive normalization tuned to reproduce subjective opinion on a large image quality dataset fails to reproduce basic cross-masking. Here we outline a solution for this problem by using artificial stimuli and by proposing a modification that makes the model easier to tune. Then, we show that the modified model is still competitive in the large-scale database. Our simulations with these artificial stimuli show that when using steerable wavelets, the conventional unit norm Gaussian kernels in divisive normalization should be multiplied by high-pass filters to reproduce basic trends in masking. Basic visual phenomena may be misrepresented in large natural image datasets but this can be solved with model-interpretable stimuli. This is an additional argument *in praise of artifice* in line with Rust and Movshon (2005).

## 1. Introduction

In the age of *big data* one may think that machine learning applied to representative databases will automatically lead to accurate models of the problem at hand. For instance, the problem of modeling the perceptual difference between images showed up in the discussion of eventual challenges at the NIPS-11 *Metric Learning* Workshop (Shakhnarovich et al., 2011). However, despite its interesting implications in visual neuroscience, the subjective metric of the image space was dismissed as a *trivial* regression problem because there are subjectively-rated image quality databases that can be used as training set for supervised learning.

Subjective image and video quality databases (such as VQEG, LIVE, TID, CID, CSIQ)^1^ certainly are a major source of raw data on how the visual system works in *naturalistic environments*. These databases describe the sensitivity of many observers to a wide range of distortions (of different nature and with different suprathreshold intensities) seen on top of a variety of natural images. These databases seem to open a number of possibilities to check the models in realistic scenarios.

Following a tradition that links the image quality assessment problem in engineering with human visual system models (Sakrison, 1977; Watson, 1993; Wang and Bovik, 2009; Bodrogi et al., 2016), these subjectively rated image databases have been used to fit models coming from classical psychophysics or physiology (Watson and Malo, 2002; Laparra et al., 2010; Malo and Laparra, 2010; Bertalmio et al., 2017). Given the similarity between these biological models (Carandini and Heeger, 2012) and feed-forward convolutional neural nets (Goodfellow et al., 2016), an interesting analogy is possible. Fitting the biological models to reproduce the opinion of the observers in the database is algorithmically equivalent to the learning stage in deep networks. This deep-learning-like use of the databases is a convenient way to train a physiologically-founded architecture to reproduce a psychophysical goal (Berardino et al., 2017; Laparra et al., 2017; Martinez-Garcia et al., 2018). When using these biologically-founded approaches, the parameters found have a straightforward interpretation as for instance the frequency bandwidth of the system or the extent of the interaction between sensors tuned to different features.

On the other hand, pure machine-learning (data-driven) approaches have also been used to predict subjective opinion. In this case, after extracting features with reasonable statistical meaning or perceptual inspiration, generic regression techniques are applied (Moorthy and Bovik, 2010, 2011; Saad et al., 2010, 2012, 2014), even though this regression has no biological grounds.

### 1.1. Eventual Problems With Databases

The problem with the above uses of naturalistic image databases is the conventional concern about training sets in machine learning: *is the training set a balanced representation of the range of behaviors to be explained?*

If it is not the case, the resulting model may be biased by the dataset and it will have generalization problems. This overfitting risk has been recognized by the authors of image quality metrics based on generic regression (Saad et al., 2012). Perceptually meaningful architectures impose certain constraints on the flexibility of the model, as opposed to generic regressors. These constraints could be seen as a sort of *Occam's Razor* in favor of lower-dimensional models. However, even in the biologically meaningful cases, there is a risk that the model found by fitting the naturalistic database misses well-known texture perception facts.

Accordingly, Laparra et al. (2010) and Malo and Laparra (2010) used artificial stimuli after the learning stage to check the Contrast Sensitivity Function and some properties of *visual masking*. Similarly, in Ma et al. (2018) after training the deep network in the dataset they have to show model-related stimuli to human observers to check if the results are meaningful (and discard eventual over-fitting).

### 1.2. The Regression Hypothesis Questioned

In this work we question the hypothesis suggested at the NIPS Metric Learning Workshop (Shakhnarovich et al., 2011) that assumes that pure regression on naturalistic databases will lead to sensible vision models.

Of course, training whatever regression model with subjectively rated natural images to predict human opinion is a *perfectly fine* approach to tackle the restricted image quality problem. Actually, sometimes disregarding any prior knowledge about how the visual system works is seen as a plus (Bosse et al., 2018): the quantitative solution to this specific problem may gain nothing from understanding the elements of a successful regression model in terms of properties of actual vision mechanisms.

However, from a broader perspective, models intended to understand the behavior of the visual system should be more ambitious: they should be interpretable in terms of the underlying mechanisms and be able to reproduce other behavior. Our message here is that large-scale naturalistic databases should not be the only source of information when trying to fit *vision models*. Given the high dimensionality of the image space, it is very likely that some basic phenomena (e.g., the visibility of certain distortions in certain environments) are under-represented in the database. As a result, the model is not forced to reproduce these under-represented phenomena. And more importantly, the use of model-interpretable artificial stimuli is useful to determine the values of specific parameters in the model.

In particular, we study a specific example of the generalization risk suggested above and the benefits of model-based artificial stimuli. We show that a wavelet+divisive normalization layer of a standard cascade of linear+nonlinear layers fitted to maximize the correlation with subjective opinion on a large image quality database (Martinez-Garcia et al., 2018), fails to reproduce basic cross-masking. Here we point out the problem and we outline a solution using well selected artificial stimuli. Then, we show that the model corrected to account for these extra artificial tests is also a competitive explanation for the large-scale naturalistic database. This example is interesting because showing convincing Maximum Differentiation stimuli, as done in Berardino et al. (2017), Martinez-Garcia et al. (2018), and Ma et al. (2018), may not be enough to guarantee that the model reproduces related behaviors and points out the need to explicitly check with artificial stimuli.

### 1.3. In Praise of Artifice: Interpretable Models and Interpretable Stimuli

In line with Rust and Movshon (2005), our results in this work, namely pointing out the misrepresentation of basic visual phenomena in subjectively-rated natural image databases and the proposed procedure to fix it, are additional arguments *in praise of artifice*: the artificial model-motivated stimuli in classical visual neuroscience are helpful to (a) point out the problems that remain in models fitted to natural image databases, and (b) to suggest intuitive modifications of the models.

Regarding interpretable models, we propose a modification for the considered Divisive Normalization (Carandini and Heeger, 2012) that stabilizes its behavior. As a result of this stabilization, the model is easy to tune (even by hand) to qualitatively reproduce cross-masking. Interestingly, as a consequence of this modification and analysis with artificial stimuli, we show that the conventional unit-norm kernels in divisive normalization may have to be re-weighted depending on the selected wavelets.

It is important to note that the observations made in this work are not restricted to the specific image quality problem. Following seminal ideas based on information theory (Attneave, 1954; Barlow, 1959), theoretical neuroscience considers explanations of sensory systems based on statistical learning as alternative to physiological and psychophysical descriptions (Dayan and Abbott, 2005). Therefore, the points made below on natural image datasets, artificial stimuli from interpretable models, and optimization goals in statistical learning, also apply to a wider range of computational explanations.

The paper is organized as follows: section 2 describes the visual stimuli and introduces the cortical models considered in the work. First it illustrates the intuition that can be obtained from proper artificial stimuli as opposed to the not-so-obvious interpretation of natural stimuli. Then, it presents the structure of wavelet-like responses in V1 cortex and two standard neural interaction models: **Model A** (intra-band), and **Model B** (inter-band). Section 3 shows that despite **Model A** is tuned to maximize the correlation with subjective opinion in a large-scale naturalistic image quality database it fails to reproduce basic properties of visual masking. Simulations with artificial stimuli allow intuitive tuning of **Model B** to get the correct contrast response curves while preserving the success on the large-scale naturalistic database. Finally, as suggested by the failure-and-solution example considered in this work, in section 4 we discuss the opportunities and precautions of the use of natural image databases to fit vision models, and the relevance of artificial stimuli based on interpretable models.

## 2. Materials and Methods

Here we present the visual stimuli and the cortical interaction models considered throughout the work. The use of model-inspired artificial stimuli is critical to point out the limitations of simple models and to tune the parameters of more general models.

### 2.1. Natural vs. Artificial Stimuli

**Figure 1:**
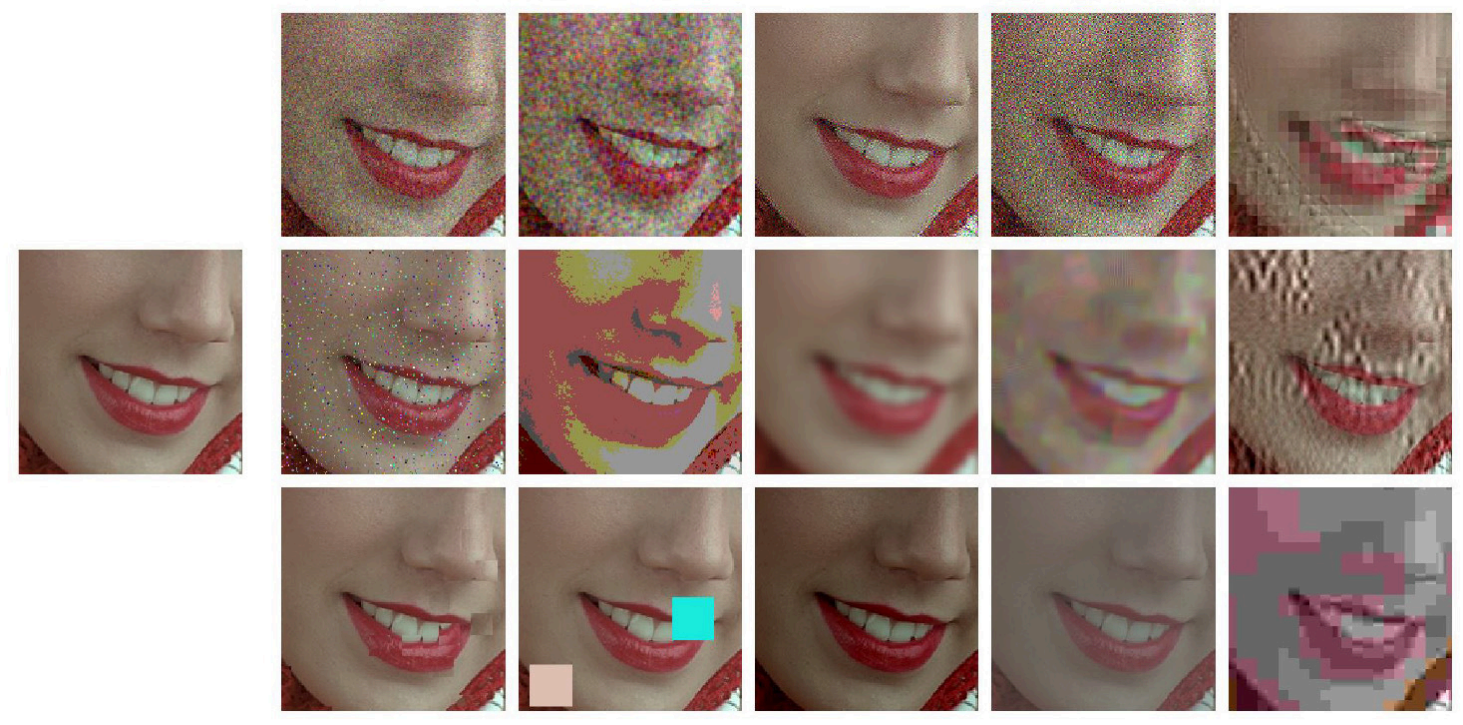
Natural scenarios and complex distortions. The isolated image at the left is an example of a natural background (uncontrolled scene) to be distorted by a variety of degradations of different nature. The images in the array illustrate the kind of stimuli rated by the observers in image quality databases. The score of the degraded images is related to the visibility of the corresponding distortion (the test) seen on top of the original image (the background). The reported subjective ratings constitute the ground truth that should be predicted by vision models from the variation of the responses due to the distortions.

Figure 1 shows a representative subset of the kind of patterns subjectively rated in image quality databases. This specific example comes from the TID2008 database (Ponomarenko et al., 2008). In these databases, natural scenes (photographic images with uncontrolled content) are corrupted by noise sources of different nature. Some of the noise sources are stationary and signal independent, while others are spatially variant and depend on the background. Ratings depend on the visibility of the distortion seen on top of the natural background. The considered distortions come in different suprathreshold intensities. In some cases these intensities have controlled (linearly spaced) energy or contrast, but in general, they come from arbitrary scales. Examples include different compression ratio or color quantization coarseness with no obvious psychophysical meaning. This is because the motivation of the original databases (e.g., VQEG or LIVE) was the assessment of distortions occurring in *image processing* applications (e.g., transmission errors in digital communication) and not necessarily to be a tool for *vision science*. More recent databases include more accurate control of luminance and color of both the backgrounds and the distortions (Pedersen, 2015), or report the intensities of the distortions in JND units (Alam et al., 2014). Perceptual ratings in such diverse sets certainly provide a great ground truth to check vision science models in naturalistic conditions.

However, the result of such variety is that the backgrounds and the tests seen on top have no clear interpretation in terms of specific perceptual mechanisms or controlled statistics in a representation with physiological meaning. Even though not specifically directed against subjectively rated databases, this was also the main drawback pointed out in Rust and Movshon (2005) against the use of generic natural images in vision science experiments.

In this work we go a step further in that criticism: due to the uncontrolled nature of the natural scenes and the somewhat arbitrary distortions found in these databases, the different aspects of a specific perceptual phenomenon are not fully represented in the database. Therefore, these databases should be used carefully when training models because this misrepresentation will have consequences when fitting the models.

For instance, let's consider pattern masking (Foley, 1994; Watson and Solomon, 1997). It is true that some distortions in the databases introduce relatively more noise in high contrast regions, which seems appropriate to illustrate masking. This is the case of the JPEG or JPEG2000 artifacts, or the so called *masked noise* in the TID database. See for instance the third example in the first row of Figure 1. These deviations on top of high contrast regions are less visible than equivalent deviations of the same energy on top of flat backgrounds. This difference in visibility is due to the inhibitory effect of surround in *masking* (Foley, 1994; Watson and Solomon, 1997). Actually, perceptual improvements of image coding standards critically depend on using better masking models that allow using less bits in those regions (Malo et al., 2000a, 2001, 2006; Taubman and Marcellin, 2001). Appropriate prediction of the visibility of these distortions in the database should come from an accurate model of texture masking. However, a systematic set of examples illustrating the different aspects of masking is certainly not present in the databases. For example, there are no stimuli showing crossmasking between different frequencies in different backgrounds. Therefore, this phenomenon is under-represented in the database.

**Figure 2:**
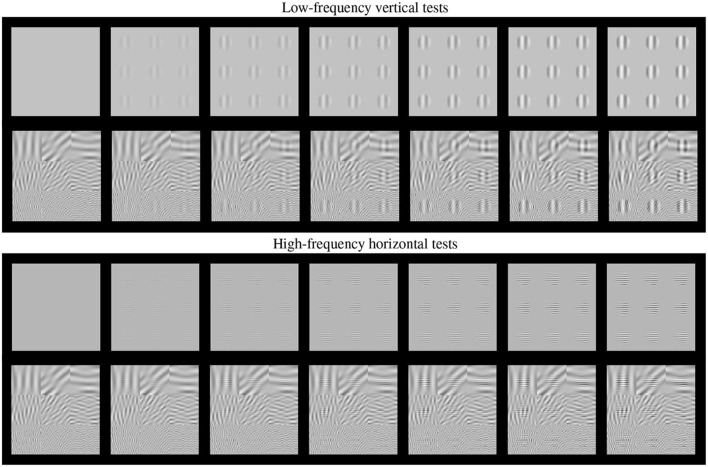
Artificial stimuli. Several texture phenomena illustrated in a single figure (see text for details). Here the tests are the 9 patterns in the gray frames. These tests increase in contrast from the frame at the left to the frame at the right. The visibility of the tests (a) nonlinearly increases with the contrast from left to right; (b) the visibility depends on the frequency of the tests, low frequency at the top panel and high frequency at the bottom panel; and (c) the visibility of the tests depends on the background (cross-masking).

Such basic texture perception facts can be easily illustrated using artificial stimuli. Artificial stimuli can be designed with a specific perceptual phenomenon in mind, and using patterns which have specific consequences in models, e.g., stimulation of certain sensors of the model. Model/phenomenon-based stimuli is the standard way in classical psychophysics and physiology. Figure 2 is an example of the power of well controlled artificial stimuli: it represents a number of major texture perception phenomena in a single figure.

This figure shows two basic tests (low-frequency vertical and high-frequency horizontal) of increasing contrast from left to right. These series of tests are, respectively, shown on top of (a) no background, and (b) on top of backgrounds of controlled frequency and orientation.

First, of course we can see that the visibility of the tests (or the response of the mechanisms that mediate visibility) increases with contrast, from left to right. This is why even the trivial Euclidean distance between the original and the distorted images is positively correlated with subjective opinion of distortion.

Second, the visibility, or the responses, depend(s) on the frequency of the test. Note that the lower frequency test is more visible than the high frequency test at reading distance. This illustrates the effect of the Contrast Sensitivity Function (Campbell and Robson, 1968).

Third, the response increase is non-linear with contrast. Note that for lower contrasts (e.g., from the second picture to the third in the series) the increase in visibility is bigger than for higher contrasts (e.g., between the pictures at the right-end). This means that the slope of the mechanisms mediating the response is high for lower amplitudes and saturates afterwards. This sort of Weber-like behavior for contrast is a distinct feature of contrast masking (Legge, 1981).

Finally, the visibility (or response) decreases with the background energy depending on the spatio-frequency similarity between test and background. Note for instance that the low frequency test is less visible on top of the low frequency background than on top of the high frequency background. Important for the example considered throughout this paper, note that the visibility of the high frequency test behaves *the other way around*: it is bigger on top of the low frequency test. Moreover, this *masking* effect is bigger for bigger contrasts of the background. This adaptivity of the nonlinearity is a distinct feature of the *masking* effect (Foley, 1994; Watson and Solomon, 1997), and more importantly, it is a distinct feature of real neurons (Carandini and Heeger, 1994, 2012) with regard to the simplified neurons used in deep learning (Goodfellow et al., 2016).

**Figure 3:**
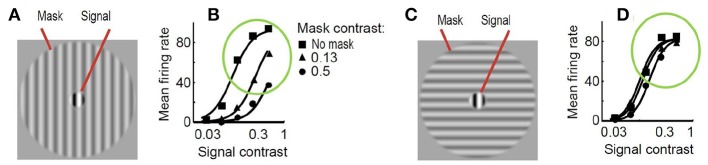
Experimental response of V1 neurons (mean firing rate) in masking situations. Adapted from (Cavanaugh, 2000; Schwartz and Simoncelli, 2001). At the left **(A)** test and mask do have the same spatio-frequency characteristics. At the right **(C)** test is substantially different from the mask. Note the decay in the responses, compare the curves in green circles, when test and background share properties **(B)** as opposed to the case where they do not **(D)**.

As a result, just by looking at Figure 2, one may imagine how the visibility (or response) curves vs. the contrast of the test should be for the series of stimuli presented. Figure 3 shows an experimental example of the kind of response curves obtained in actual neurons in masking situations. Note the saturation of the response curves and how they are attenuated when the background is similar to the test. Even this qualitative behavior highlighted in green (saturation and attenuation) may be used to discard models that do not reproduce the expected behavior, i.e., that do not agree with what we are seeing.

**Figure 4:**
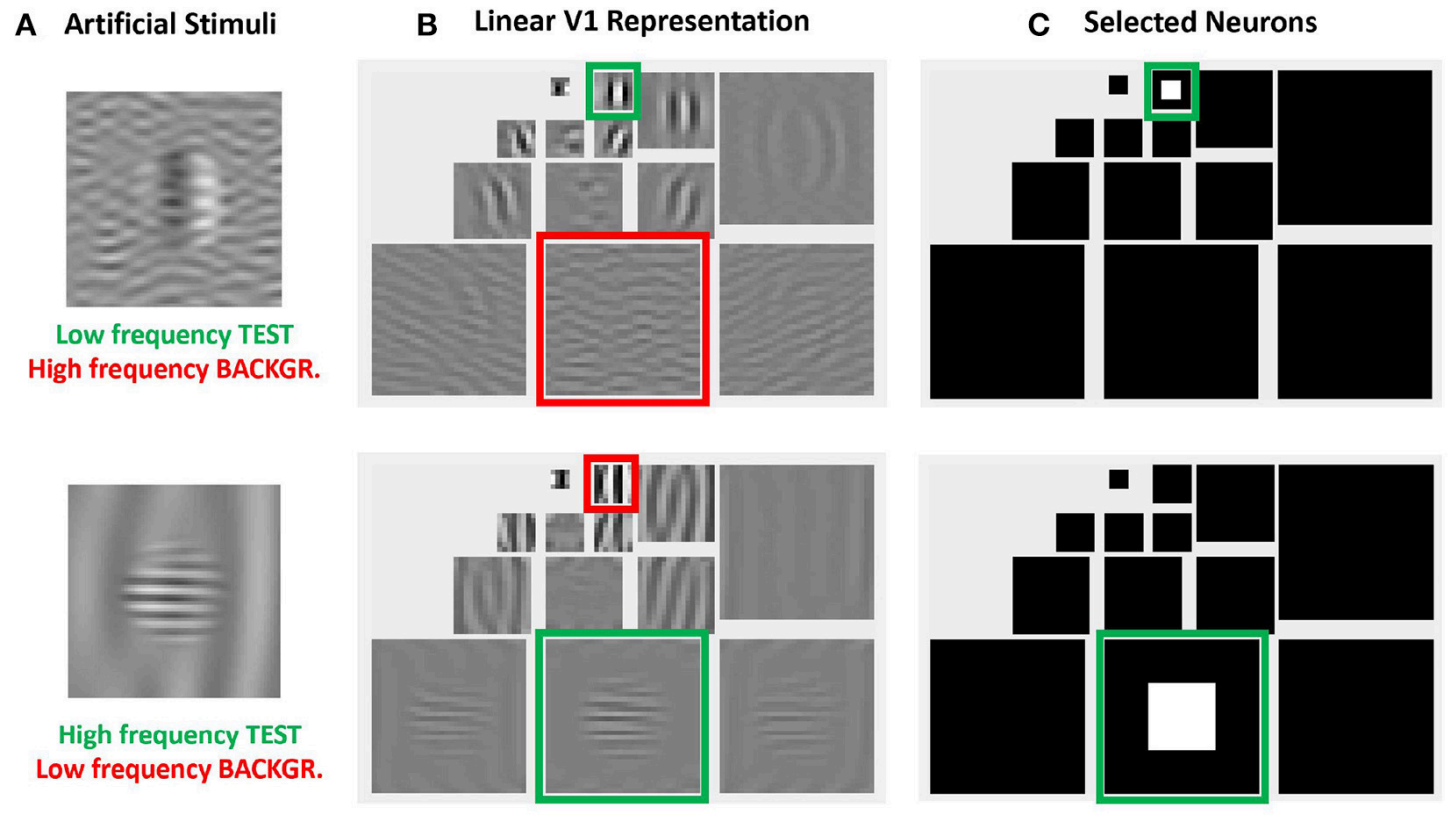
Advantages of artificial stimuli. Model-related construction of stimuli simplifies the reproduction of results form model outputs and the interpretation of results. In this example, frequency and orientation of tests and backgrounds in the artificial stimuli at the left **(A)** are selected to stimulate specific subbands of the model, see highlighted regions in the wavelet transform at the central panel **(B)**. Therefore, it is easy to select the sensors that mediate the visibility of the tests, see the coefficients in white in the wavelet diagram at the right panel **(C)**.

More importantly, the relative visibility of these artificial stimuli can also be used to intuitively tune the parameters of a model to better reproduce the visible behavior. This can be done because these artificial stimuli were crafted to have a clear interpretation in a standard model of texture vision: a set of V1-like wavelet neurons (oriented receptive fields tuned to different frequency scales). Figure 4 illustrates this fact: note how the test patterns considered in the figure mainly stimulate a specific subband of a 3-scale 4-orientation steerable wavelet pyramid (Simoncelli et al., 1992), which is a commonly used model of V1 sensors. As a result, it is easy to select the set of sensors that will drive the visibility descriptor in the model: see the highlighted wavelet coefficients in the diagrams at the right of Figure 4.

The same intuitive energy distribution over the pyramid is true for the backgrounds, which stimulate the corresponding subband (scale and orientation). As a result, given the distribution of test and backgrounds in the pyramid, it is easy to propose intuitive cross-band inhibition schemes to lead to the required decays in the response.

The intuitions obtained from artificial model-oriented stimuli about response curves and eventual-crossmasking schemes are fundamental both to criticize the results obtained from *blind learning from a database*, and to propose intuitive improvements of the model.

### 2.2. Cortical Interaction Models: Structure and Response

In this work we analyze the behavior of standard retina-cortex models that follow the program suggested in Carandini and Heeger (2012) i.e., cascades of isomorphic linear+nonlinear layers, each focused on a different psychophysical factor:

Layer *S*^(1)^ linear spectral integration to compute luminance and opponent tristimulus channels, and nonlinear brightness/color response.
Layer *S*^(2)^ definition of local contrast by using linear filters and divisive normalization.
Layer *S*^(3)^ linear LGN-like contrast sensitivity filter and nonlinear local energy masking in the spatial domain.
Layer *S*^(4)^ linear V1-like wavelet decomposition and nonlinear divisive normalization to account for orientation and scale-dependent masking.

This family of models represents a system, *S*, that depends on some parameters, **Θ**, and applies a series of transforms on the input radiance vector, ***x***^0^, to get a series of intermediate response vectors, ***x***^*i*^,

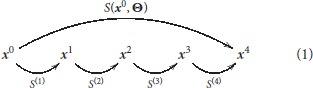



Each layer in this sequence accounts for the corresponding psychophysical phenomenon outlined above and is the concatenation of a linear transform L and a nonlinear transform N:

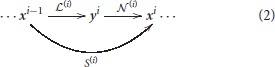



Here, in each layer we use convolutional filters for the linear part and the canonical Divisive Normalization for the nonlinear part. The mathematics of this type of models required to set their parameters are detailed in Martinez-Garcia et al. (2018).

In this kind of models the psychophysical behavior (visibility of a test) is obtained from the behavior of individual units (increment of responses) through some sort of *summation*. The visibility of a test, Δ***x***^0^, seen on top of a background, ***x***^0^, is given by the perceptual distance between *background* and *background+test*. Specifically, this perceptual distance, *d*_*p*_, may be computed through the *q* norm of the vector with the increment of responses in the last neural layer (Watson and Solomon, 1997; Laparra et al., 2010; Martinez-Garcia et al., 2018). In the 4-layer model of Equation 1, we have $\|\Delta x^{4}{\|}_{q}$:

(3) $$\begin{array}{l} d_{p}\left(x^{0},x^{0}+\Delta x^{0}\right)=\|\Delta x^{4}{\|}_{q}={\left(\underset{j}{\sum }|\Delta x_{j}^{4}{|}^{q}\right)}^{\frac{1}{q}} \end{array}$$

There is a variety of summation schemes: one may choose to use different summation exponents for different features (e.g., splitting the sum over *j* in space, frequency, and orientation), and order of summation matters if the exponents for the different features are not the same. Besides, there is no clear consensus on the value of the summation exponents either (Graham, 1989): the default quadratic summation choice, *q* = 2 (Teo and Heeger, 1994; Martinez-Garcia et al., 2018), has been questioned proposing bigger (Watson and Solomon, 1997; Laparra et al., 2010) and smaller (Laparra et al., 2017) summation exponents.

More important than all the above technicalities, the key points in Equation (3) are: (a) it clearly relates the visibility with the response of the units, and (b) for *q* ≥ 2, the visibility is driven by the response of the units that undergo bigger variation, $\left|\Delta x_{j}^{4}\right|$, such as the ones highlighted in Figure 4. Therefore, in this kind of models, analyzing the visibility curves or the response curves of the units tuned to the test is qualitatively the same. In the simulations we do the latter since we are interested in direct observation of the effect of the interaction parameters on the curves; and this is more clear when looking at the response of selected subsets of units as those highlighted in Figure 4.

In this work we compare two specific examples of this family of models. These two models will be referred to as **Model A** and **Model B**. They have identical layers 1–3, and they only differ in the nonlinear part of the fourth layer: the stage describing the interaction between cortical oriented receptive fields. In **Model A** we only consider interactions between the sensors tuned to the same subband (scale and orientation) because we proved that this simple scheme is appropriate to obtain good performance in subjectively rated databases (Laparra et al., 2010; Malo and Laparra, 2010). In **Model B** on top of the intra-band relation we also considered inter-band relations according to a standard unit-norm Gaussian kernel over space, scale and orientation (Watson and Solomon, 1997). Additionally to the classical inter-band generalization we also included extra weights and a stabilization constant that makes the model easier to understand. The software implementing **Model A** and **Model B** is available at “http://isp.uv.es/docs/BioMultiLayer_L_NL_a_and_b.zip”.

Let's consider the differences between the models in more detail. Assuming that the output of the wavelet filter-bank is the vector ***y***, and assuming that the vector of energies of the coefficients is obtained by coefficient-wise rectification and exponentiation, ***e*** = |***y***|^γ^, the vector of responses after divisive normalization in the last layer of **Model A** is:

(4) $$\begin{array}{l} x=sign\left(y\right)⊙\frac{e}{b+H\cdot e} \end{array}$$

where ⊙ stands for element-wise Hadamard product and the division is also an element-wise Hadamard quotient where the energy of each linear response is divided by a linear combination of the energies of the neighboring coefficients in the wavelet pyramid. This linear combination (that attenuates the response) is given by the matrix-on-vector product *H* · ***e***. Note that, for simplicity, in Equation 4 we omitted the indices referring to the 4th layer [as opposed to the more verbose formulation in the Appendix (Supplementary Material)].

The *i*-th row of this matrix, *H*, tells us how the responses of neighbor sensors in the vector ***e*** attenuate the response of the *i*-th sensor in the numerator, *e*_*i*_. The attenuating effect of these linear combinations is moderated by the semisaturation constants in vector ***b***.

**Figure 5:**
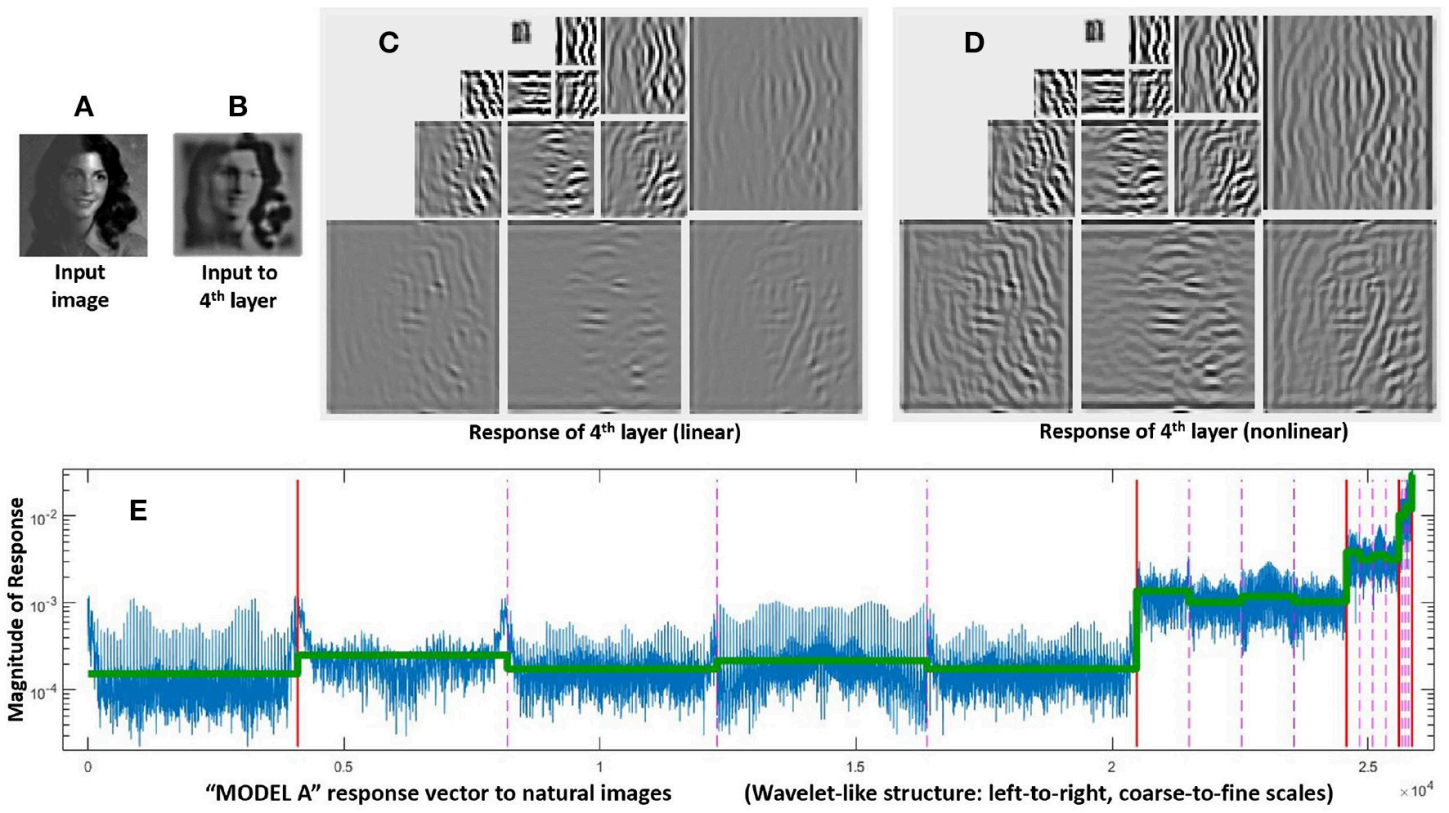
Response of Model A to natural images. Given a luminance distribution, input image **(A)**, the initial layers of the model (retina-to-LGN) compute a filtered version of brightness contrast with adaptation to lower contrasts due to divisive normalization. That is why the contrast in the input to the 4th layer, image **(B)**, is more uniform than in the input image. Finally, the linear part of the 4th layer, wavelet diagram **(C)**, computes a multi-scale / multi-orientation decomposition and then, these responses nonlinearly interact as given by Equation (4), final responses in wavelet diagram **(D)**. The structure of a representative vector of responses depicted at the bottom is relevant to understand the assumed interactions and the eventual modifications that may be required. As usual in the wavelet literature (Simoncelli and Adelson, 1990), data in the vector are organized from high-frequency (fine scales at the left) to low-frequency (coarse scales at the right), wavelet vector **(E)**. Abscissas indicate the wavelet coefficient. The specific scale of the ordinate axis is not relevant. Solid vertical lines in red indicate the limits of the different scales. Within each scale, the dashed lines in pink indicate the limits of the different orientations. The different coefficients within each scale/orientation block correspond to different spatial locations. The line in green shows the average amplitude per subband for a set of natural images. As discussed in the text, this specific energy distribution per scale is relevant for the good performance of the model.

The structure of these vectors and matrices is relevant to understand the behavior on the stimuli. First, one must consider that all the vectors, ***y***, ***e***, and ***x***, have wavelet-like structure. Figure 4 shows this subband structure for specific artificial stimuli and Figure 5 shows it for natural stimuli.

The *i*-th coefficient has a 4-dimensional spatio-frequency meaning, *i* ≡ (***p***_*i*_, *f*_*i*_, ϕ_*i*_), where ***p*** is a two-dimensional location, *f* is the modulus of the spatial frequency, and ϕ is orientation.

In **Model A** we only consider Gaussian intra-band relations. This means that interactions in *H* decay with spatial distance and it is zero between sensors tuned to different frequency and orientation. This implies a block-diagonal structure in *H* with zeros in the off-diagonal blocks. In Martinez-Garcia et al. (2018) the norm or each Gaussian neighborhood (or row) in *H* was optimized to maximize the correlation with subjective opinion.

It is important to stress that the specific distribution of responses of natural images over the subbands of the response vector (green line in Figure 5) is critical to reproduce the good behavior of the model on the database. Note that this is not a regular (linear) wavelet transform, but the (nonlinear) response vector. Therefore, this distribution tells us *both* about the statistics of natural images and about the behavior of the visual system. On the one hand, natural images have relatively more energy in the low-frequency end. But, on the other hand, it is visually relevant that the response of sensors tuned to the high frequency details is much lower than the response of the sensors tuned to the low frequency details. The latter is in line with the different visibility of the artificial stimuli of different frequency shown in Figure 2, and it is probably due to the effect of the Contrast Sensitivity Function (CSF) in earlier stages of the model. This is important because keeping this relative magnitude between subbands is crucial to have good alignment with subjective opinion in the large-scale database.

In the case of **Model B**, we consider (a) a more general interaction kernel in the divisive normalization, and (b) a constant diagonal matrix to control the dynamic range of the responses. Specifically, the vector of responses is:

(5) $$\begin{array}{l} x=sign\left(y\right)⊙\left[\kappa ⊙\frac{b+H_{G}\cdot e^{⋆}}{e^{⋆}}\right]⊙\frac{e}{b+H_{G}\cdot e}. \end{array}$$

Here the response still follows a nonlinear divisive normalization because ***e***^⋆^ is just a fixed vector (not a variable), and hence the term in brackets is just another constant vector. In **Model B**, following Watson and Solomon (1997), we consider a generalized interaction kernel *H*_*G*_ that consists of separable Gaussian functions which depend on the distance between the location of the sensors, *H*_***p***_, and on the difference between their scales and orientations, *H*_*f*_ and *H*_ϕ_. Moreover, we extend the unit-norm Gaussian kernel already proposed in Watson and Solomon (1997) with additional weights in case extra inter-band tuning is needed:

(6) $$\begin{array}{l} H_{G}={𝔻}_{c}\cdot \left[H_{p}⊙H_{f}⊙H_{\phi }⊙C_{\text{int}}\right]\cdot {𝔻}_{w}, \end{array}$$

where *C*_int_ is a subband-wise full matrix, 𝔻_***w***_ is a diagonal matrix with vector ***w*** in the diagonal, and the normalization of each row of the kernel is controlled by a diagonal matrix 𝔻_***c***_, which contains the vector of normalization constants, ***c***, in the diagonal. This means that the elements *c*_*i*_ normalize each interaction neighborhood, and the elements *w*_*j*_ control the relative relevance of the energies *e*_*j*_ before these are considered for the interaction.

In addition to the generalized kernel, the other distinct difference of **Model B** is the extra constant $K\left(e^{⋆}\right)=\left[\kappa ⊙\frac{b+H_{G}\cdot e^{⋆}}{e^{⋆}}\right]$. This constant has a relevant qualitative rationale: it keeps the response bounded *regardless of the choice for the other parameters*.

Note that, when the input energy, ***e***, arrives to the *reference value*, ***e***^⋆^, the response of **Model B** reduces to the vector ***κ*** regardless of model parameters. This simplifies the qualitative control of the dynamic range of the system because one may set a desired output ***κ*** (e.g., certain amplitudes per subband) for some relevant reference input ***e***^⋆^ regardless of the other parameters. This stabilization constant, *K*(***e***^⋆^), does not modify the qualitative effect of the relevant parameters of the divisive normalization, but, as it constraints the dynamic range, it allows the modeler to freely play with the relevant parameters γ, ***b***, and *H*_*G*_, and still preserve the relative amplitude of the subbands. And this freedom is particularly critical to understand the kind of modifications needed in the parameters to reproduce certain experimental trend.

Here we propose that ***e***^⋆^ is related to the average energy of the *input* to this nonlinear neural layer. Similarly, we propose to set the global scaling factor, ***κ***, according to a desired dynamic range in the *output* of this neural layer. These stabilization settings simplify the use of the model thus allowing to get the desired qualitative behavior even modifying the parameters *by hand*. Interestingly, this freedom to explore will reveal the modulation required in the conventional unit-norm Gaussian kernel.

## 3. Results

In this section we show the performance of **Model A** and **Model B** in two scenarios: (a) reproducing subjective opinion in large-scale naturalistic databases using quadratic summation in Equation 3, and (b) obtaining meaningful contrast response curves for artificial stimuli.

The parameters of **Model A** are those obtained in Martinez-Garcia et al. (2018) to provide the best possible fit to the mean opinion scores on a large natural image database. These parameters of **Model A** are kept fixed throughout the simulations in this section. On the contrary, in the case of **Model B**, we start from a base-line situation in which we import the parameters from **Model A**, but afterwards, this naive guess is fine tuned to get reasonable response curves for the artificial stimuli considered above. Our goal is checking if the models account for the trends of masking described in Figures 2, 3: we are not fitting actual experimental data but just refuting models that do not follow the qualitative trend.

In this model verification context, the fine tuning of **Model B** is done *by hand*: we just want to stress that while **Model A** cannot account for specific inter-band interactions, the interpretability of **Model B** when using the proper artificial stimuli makes it very easy to tune. And this intuitive tuning is possible thanks to the stabilization effect of the constant *K*(***e***^⋆^) proposed above.

Nevertheless, it is important to stress that the Jacobian with regard to the parameters of **Model B** given in appendix (Supplementary Material) are implemented in the code associated to the paper. Therefore, despite the exploration of the responses in this section will be just qualitative, the code of **Model B** is ready for gradient descent tuning if one decides to measure the contrast incremental thresholds for the proper artificial stimuli.

Accurate control of spatial frequency, luminance, contrast and appropriate rendering of artificial stimuli can be done using the generic routines of VistaLab (Malo and Gutiérrez, 2014). In order to do so, one has to take into account a sensible sampling frequency (e.g., bigger than 60 cpd to avoid aliasing at visible frequencies) and the corresponding central frequencies and orientations of the selected wavelet filters in the model. The specific software used in this paper to generate the stimuli and to compute the response curves is available at: “http://isp.uv.es/docs/ArtificeReloaded.zip”.

### 3.1. Success of *“Model A”* in Naturalistic Databases

**Figure 6:**
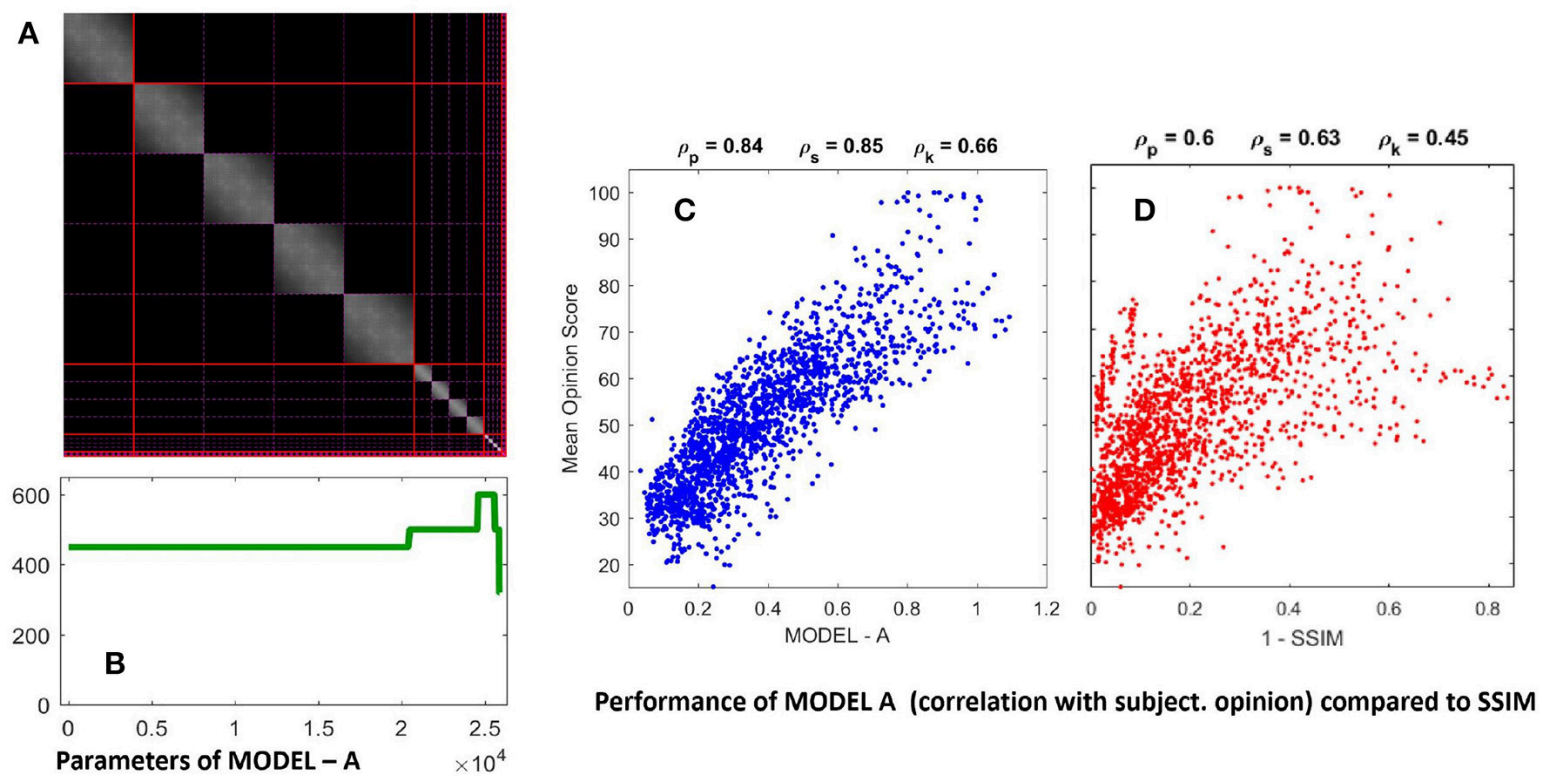
Parameters of MODEL-A (left, **A,B**) and performance on large scale naturalistic database (right, **C,D**). The parameters are: the interaction kernel *H* (matrix on top, **A**), and the semisaturation per subband vector, ***b***. The structure of ***b*** is the same as a wavelet vectors in Figure 5. The kernel *H* describes how each wavelet coefficient interacts with the others, therefore, we included the solid and dashed lines in red and pink to highlight the limits between the subbands. The resulting submatrices describe the intra- and inter-subband interactions. The figures on top of the scatter plots are the Pearson, Spearman, and Kendall correlations. Here performance of Model A in plot **(C)** is compared with SSIM (Wang et al., 2004) in **(D)** just because it is the de-facto standard in image quality assessment.

Optimization of the width and amplitude of the Gaussian kernel, *H*, in each subband as well as the semisaturation parameters ***b*** in each subband of **Model A** led to the results in Figure 6. This was referred to as *optimization phase I* in Martinez-Garcia et al. (2018). Even though *optimization phase II* using the full variability in ***b*** led to higher correlations, here we restrict ourselves to *optimization phase I* because we want to keep the number of parameters small. Note that ***b*** has 2.5·10^4^ elements but restricting to a single semisaturation per subband we only have 14 free parameters. In the *optimization phase I* only 1/25 of the TID database was used in the training.

As stated above, spatial-only intra-band relations leads to symmetric block diagonal kernels. Optimization acted on the width and amplitude of these kernels per subband. Similarly, optimization lead to bigger semisaturation for low frequencies except for the low-pass residual.

The performance of the resulting model on the naturalistic database is certainly good: compare the correlation of **Model A** with subjective opinion in Figure 6 as opposed to the widely used Structural SIMilarity index (Wang et al., 2004), in red, considered here just as useful reference. Given the improvement in correlation with regard to SSIM, one can certainly say that **Model A** is *highly successful* in predicting the visibility of uncontrolled distortions seen on naturalistic backgrounds.

### 3.2. Relative Failure of *“Model A”* With Artificial Stimuli

Despite the reasonable formulation of **Model A** and its successful performance in reproducing subjective opinion in large-scale naturalistic databases, a simple simulation with the kind of artificial stimuli presented in section 2.1 shows that it does not reproduce all the aspects of basic visual masking.

Specifically, we computed the response curves of the highlighted neurons in Figure 4 for low-frequency and high-frequency tests like those illustrated in Figure 2 as a function of their contrast. We considered four different contrasts for the background. Different orientations of the background (vertical, diagonal and horizontal) were also considered.

**Figure 7:**
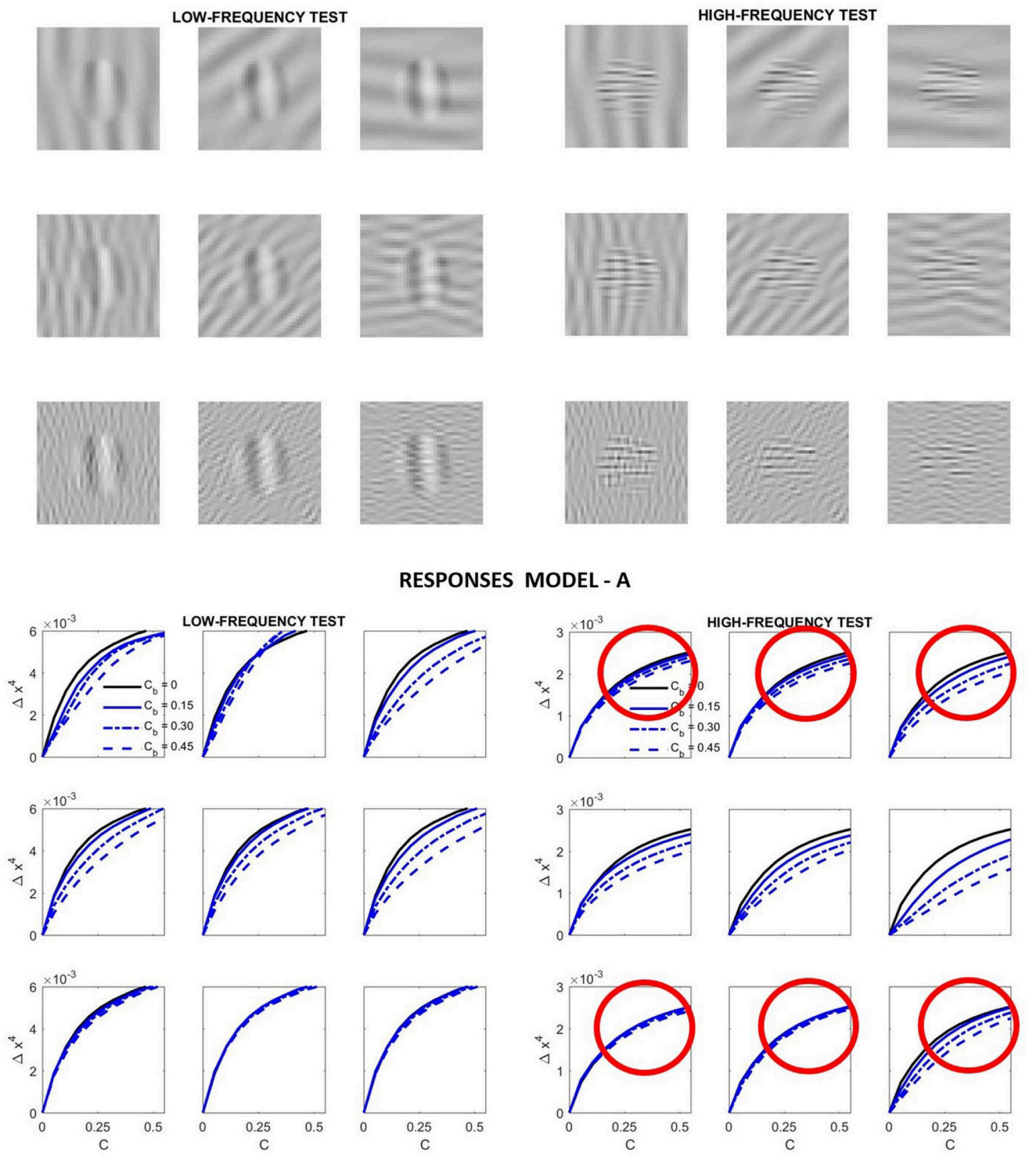
Relative success and failures of **Model A** optimized on the large-scale database. Model-related stimuli such as the low-frequency and high-frequency tests shown on the top panel simplify the reproduction of results form model outputs and allow simple visual interpretation of results. In this simulation the response curves at the bottom panel are computed from the variation of the responses of the low-frequency and high-frequency sensors of the 4th layer highlighted in green in Figure 4. In each case, the variation of the response is registered as the contrast of the corresponding stimulus is increased. That is why we plot Δ***x***^4^ vs. the contrast of the input, C. The different line styles represent the response for different contrast of the background, *C*_*b*_. Simple visual inspection of the stimuli is enough to discard some of the predicted curves (e.g., those in red circles): the low frequency backgrounds *do not* mask the high frequency test more than the high frequency backgrounds.

Figure 7 presents the results of such simulation. This figure highlights some of the good features of **Model A**, but also its shortcomings.

On the positive side we have the following. First, the response increases with contrast as expected. Second, the response for the low frequency test is bigger than the response for the high frequency test (see the scale of the ordinate axis for the high frequency response). This is in agreement with the CSF. Third, the response saturates with contrast as expected. And also, increasing the contrast of the background decreases the responses.

However, *contrarily to what we can see when looking at the artificial stimuli*, the response for the high frequency test *does not* decay more on top of high frequency backgrounds. While the decay behavior is qualitatively ok for the low-frequency test, definitely it is not ok for the high-frequency test. Compare the decays of the signal at the circles highlighted in red in Figure 7: the response of the sensors tuned to high-frequency test decays by the same amount when they are presented on top of low-frequency backgrounds than when the background also has high-frequency. The model is failing here despite its good performance in the large database.

### 3.3. Success of *“Model B”* With Natural and Artificial Stimuli

**Figure 8:**
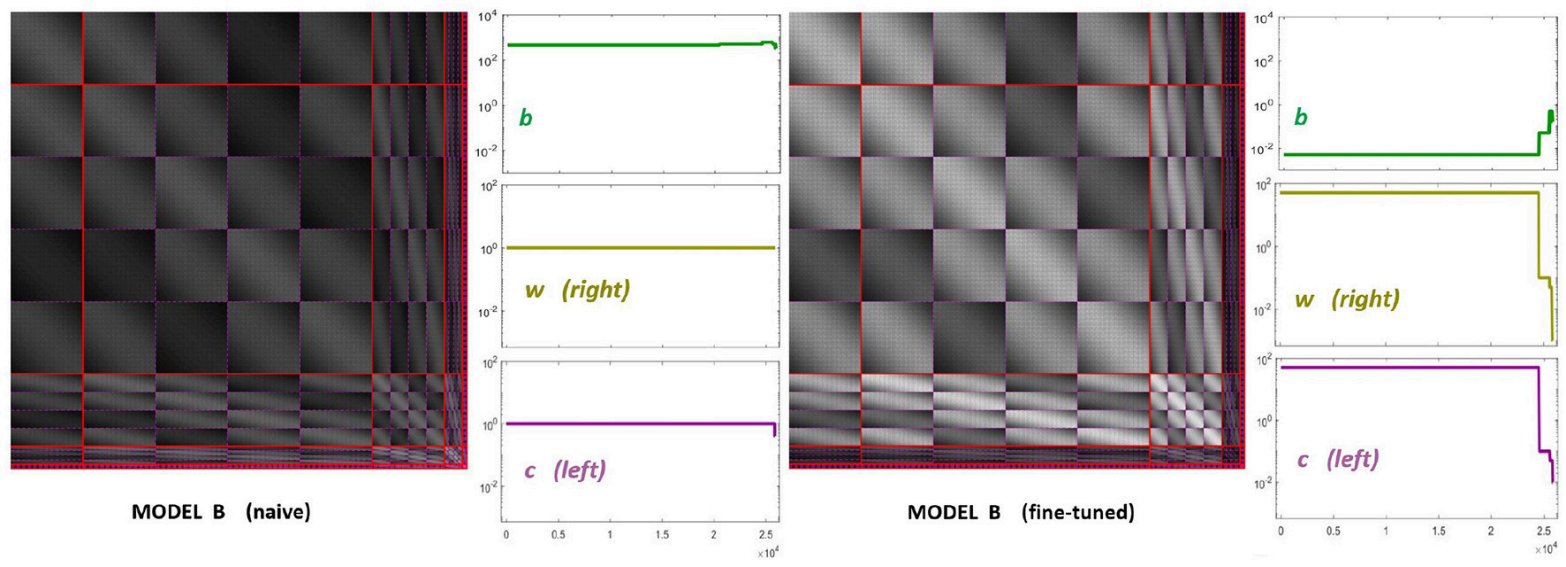
Parameters of diferent versions of **Model B**. Left panel shows the parameters for the first guess of Model B: the interaction matrix, the semisaturation vector, ***b***, and the right and left filters (vectors ***w*** and ***c*** in Equation 6). This first-guess is called *naive* because the semisaturation and amplitudes of the kernel were imported directly from **Model A** in Martinez-Garcia et al. (2018). The panel at the right shows the corresponding parameters for the fine-tuned version of Model B, once we explored a range of values to fix the response curves. See text for details on how the parameters were tuned to get the desired responses.

The starting point of our heuristic exploration with **Model B** is a straightforward translation of **Model A** into **Model B**. We will refer to this as **Model B naive**. This starting point consists of importing the values of the parameters from **Model A** except for the modulations depending on the scale and orientation. Following Watson and Solomon (1997) we assumed reasonable interaction lengths of one octave (for scales) and 30 degrees (for orientation). We used no extra weights to break the symmetry (*C*_int_ = 𝟙 is an all-ones matrix, and *C*_***w***_ = *I* is the identity). And the values for ***c*** and ***b*** also come from **Model A**. The parameters of this **Model B naive** are shown in Figure 8 (left panels). The idea of this starting point, **Model B naive**, is reproducing the behavior of **Model A** to build on from there.

**Figure 9:**
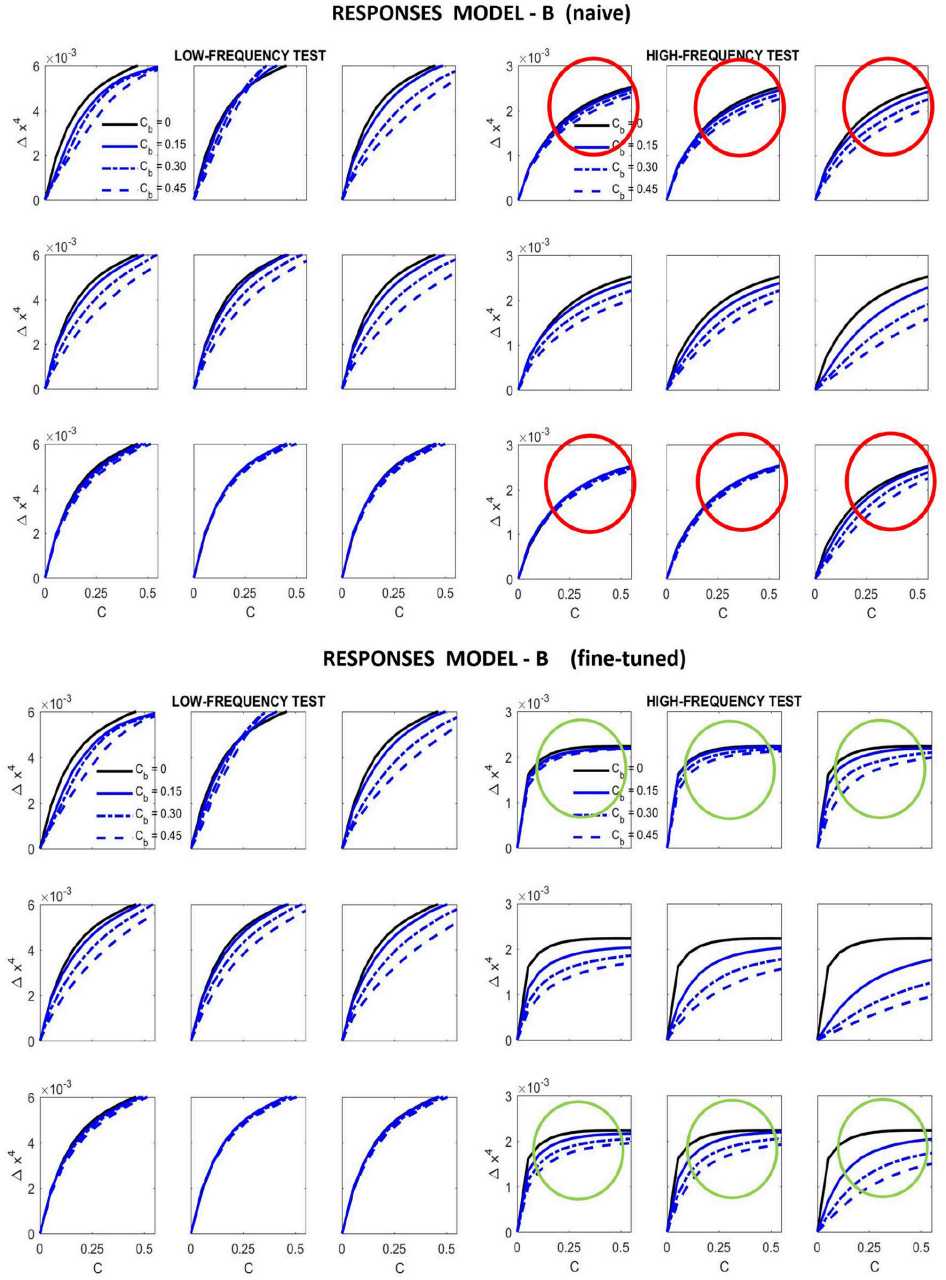
Responses of different versions of **Model B** for the artificial stimuli. Curves correspond to the same stimuli considered in Figure 7.

**Figure 10:**
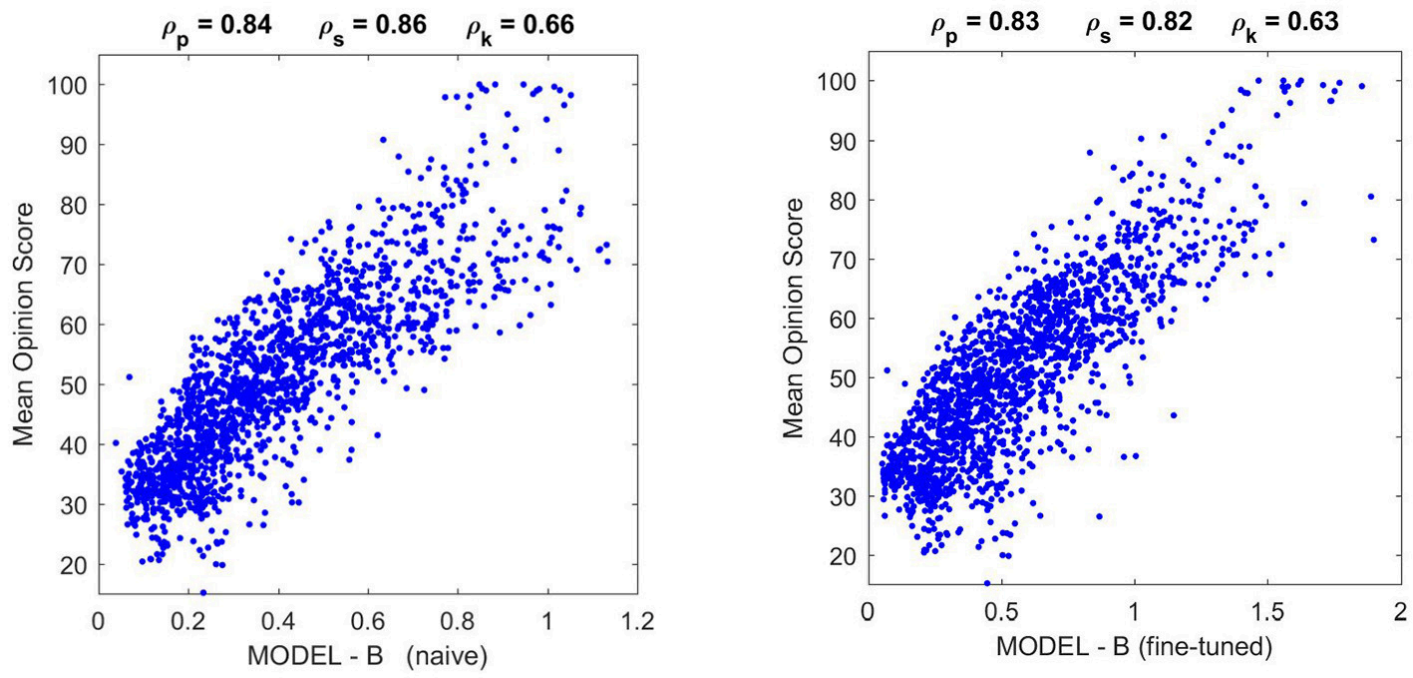
Performance of different versions of **Model B** on the natural image database. The difference in correlation is not statistically significant according to the *F*-test used in Watson and Malo (2002), and the trend of the scatter plot is qualitatively the same.

Results in Figure 9 (top) and Figure 10 (left) show that **Model B naive** certainly reproduces the behavior of **Model A**: both the success in the natural image database and the relative failure with artificial stimuli.

On top of kernel generalization, there is a second relevant intuition: modifications in the kernel may be ineffective if the semisaturation constants are too high. Note that the denominator of Divisive Normalization, Equation 4, is a balance between the linear combination *H* · ***e*** and the vector ***b***. This means that some elements of ***b*** should be reduced for the subbands where we want to act. Increasing the corresponding elements of vector ***c***, leads to a similar effect.

With these intuitions one can start playing with *H*_*G*_ and ***b***. However, while the effect of the low-frequency is easy to reduce using the above ideas (thus solving the problem highlighted in red in Figure 7), the relative amplitude between the responses to low and high frequency inputs is also easily lost. This quickly ruins the low-pass CSF-like behavior and reduces the performance on the large-scale database. We should not lose the relative amplitudes of the responses of **Model A** to natural images (i.e., green lines in Figure 5) to keep its good performance. Unfortunately **Model A** is unstable under this kind of modifications making it difficult to tune. That is why it is necessary to include the constant $\left[\kappa ⊙\frac{b+H_{G}\cdot e^{⋆}}{e^{⋆}}\right]$ in **Model B** to control the dynamic range of the responses.

Figure 8 (right panel) shows the fine-tuned parameters according to the heuristic suggested above: reduce semisaturation in certain bands and control the amplitude of the kernel in certain bands. This heuristic comes from the meaning of the blocks in the kernel and from the subbands that are activated by the different artificial stimuli. Note that we strongly reduced ***b*** and we applied bigger reductions for the high-frequency bands (which corresponds to the sensors we want to fix). In the same vein we increased the values for the global scale of the kernels of high frequencies ***c*** while reducing substantially these amplitudes for low-frequencies to preserve previous behavior, which was ok for low-frequencies. Finally, and more importantly, we moderated the effect of the low-frequencies in masking by using small weights for the low-frequency scales in ***w***, while increasing the values for high frequency. Note how this reduces the columns corresponding to the low-frequency subbands in the final kernel *H*_*G*_, and the other way around for the high-frequency scales. This implies a bigger effect of high-frequency backgrounds in the attenuation of high-frequency sensors and reduces the effect of the low-frequency.

Results in Figure 9 show that this fine-tuning fixes the qualitative problem detected in **Model A**, which was also present in **Model B naive**. We successfully modified the response of high-frequency sensors: see the decay in the green circles compared to the behavior in the red circles. Moreover, we introduced no major difference in the low-frequency responses, which already were qualitatively correct.

Moreover, Figure 10 shows that the fine-tuned version of **Model B** not only works better for artificial stimuli but it also preserves the success in the natural image database. The latter is probably due to the positive effect of setting the relative magnitude of the responses in **Model B** as in **Model A** using the appropriate *K*(***e***^⋆^) (setting the output ***κ*** for the average input ***e***^⋆^).

It is interesting to stress that the solution to get the right qualitative behavior in the responses didn't require any extra weight in *C*_int_, which remained an all-ones matrix. We only operated row-wise and column-wise with the diagonal matrices 𝔻_***c***_ and 𝔻_**w**_, respectively.

In summary, in order to fix the qualitative problems of **Model A** with masking of high-frequency patterns, the obvious use of generalized unit-norm inter-band kernels, as in Watson and Solomon (1997), was not enough: we had to consider the activation of the different subbands due to controlled artificial stimuli to tune the weights in the left- and right- diagonal matrices that modulate the unit-norm Gaussian kernels *H*_*G*_ = 𝔻_***c***_ · [*H*_***p***_ ⊙ *H*_*f*_ ⊙ *H*_ϕ_]·𝔻_***w***_. It was necessary to include high-pass filters in ***c*** and ***w*** (see Figure 8, fine-tuned) to moderate the effect of the low-frequency backgrounds on the masking of sensors tuned to high-frequencies.

The need of these extra filters can be interpreted in a interesting way: there should be a *balanced correspondence* between the linear filters and the interaction neighborhoods in the nonlinearity. Note that different choices for the filters to model the linear receptive fields in the cortex imply different energy distributions over the subbands^2^. In this situation, if the energy in certain subband is overemphasized by the choice of the filters, the interaction neighborhoods should discount this fact.

Of course, more accurate tuning of **Model B** on actual exhaustive contrast incremental data of different tests+backgrounds may lead to more sophisticated weights in *C*_int_. However, the simple toy simulation presented here using artificial stimuli with clear interpretation was enough to (a) discard **Model A**, (b) to point out the *balance problem* between the assumed linear cortical filters and the assumed interaction kernel in divisive normalization, and even (c) to propose an intuitive solution for the problem.

## 4. Discussion

The relevant question is: *is the failure of* ***Model A*** *something that we could have expected?* And the unfortunate answer is, *yes*: the failure is not surprising given the (almost necessarily) imbalanced nature of large-scale databases. Note that it is not only that **Model A** is somewhat rigid^3^, the fundamental problem is that the specific phenomenon is not present in the database with enough frequency or intensity to force the model to reproduce it in the learning stage.

Of course, this problem is hard to solve because it is not obvious to decide in advance the kind of phenomena (and the right amount of each one) that should be present in the database(s): as a result, databases are almost necessarily imbalanced and biased by the original intention of the creators of the database.

Here we made a full analysis (problem and route-to-solution) on texture masking, but note that focus on masking was just one important but arbitrary example to stress the main message. There are equivalent limitations affecting other parts of the optimized model that may come from the specific features of the database. For instance, the luminance-to-brightness transform (first layer in models A and B) is known to be strongly nonlinear and highly adaptive (Wyszecki and Stiles, 1982; Fairchild, 2013). It can be modeled using the canonical divisive normalization (Hillis and Brainard, 2005; Abrams et al., 2007) but also other alternative nonlinearities (Cyriac et al., 2016), and this nonlinearity has been shown to have relevant statistical effects (Laughlin, 1983; Laparra et al., 2012; Laparra and Malo, 2015; Kane and Bertalmio, 2016). However, when fitting layers 1st and 4th simultaneously to reproduce subjective opinion over the naturalistic database in Martinez-Garcia et al. (2018), even though we found a consistent increase in correlation, in the end, the behavior for the first layer turned out to be almost linear. The constant controlling the effect of the anchor luminance turned out to be very high. As a result, the nonlinear effect of the luminance is small. Again, one of the reasons for this result may be that the low dynamic range of the database did not require a stronger nonlinearity at the front-end given the rest of the layers. Similar effects could be obtained with the nonlinearities of color channels if the statistics is biased (MacLeod, 2003; Laparra and Malo, 2015).

The case studied here is not only a praise of artificial stimuli, but also a praise of *interpretable models*. When models are interpretable, it is easier to fix their problems from their failures on synthetic model-interpretable stimuli. For example, the solution we described here based on considering extra interaction between the sensors is not limited to *divisive* models of adaptation. Following Bertalmio et al. (2017), it may be also applied to other interpretable models such as the *subtractive* Wilson-Cowan equations (Wilson and Cowan, 1972; Bertalmio and Cowan, 2009). In this subtractive case one should tune the matrix that describes the relations between sensors. This kind of intuitive modifications in the architecture of the models would have been more difficult, if possible at all, with non-parametric data-driven methods. In fact, there is an active debate about the actual scientific gain of non-interpretable models, such as blind regression (Castelvecchi, 2016; Bohannon, 2017).

Finally, the masking curves considered in this paper also illustrate the fact that beyond the limitations of the database or the limitations of the architecture, the learning goal is also an issue. Note that, even using the same database and model, different learning goals may have different predictive power. For instance, other learning goals applied to natural images also give rise to cross-masking. Examples include information maximization (Schwartz and Simoncelli, 2001; Malo and Gutiérrez, 2006), and error minimization (Laparra and Malo, 2015). A systematic comparison between these different learning goals on the same database for a wide range of frequencies is still needed.

### 4.1. Consequence for Linear + Nonlinear Models: The Filter-Kernel Balance

Related to *model interpretability*, the results of our exploration with artificial stimuli suggests an interesting conclusion when dealing with linear+nonlinear models: *matching linear filters and non-linear interaction is not trivial*. Remember the *wavelet-kernel balance problem* described at the end of the results. Therefore, in building these models, one should not take filters and kernels off the shelf.

One may take this *balance problem* as another routinary parameter to tune. However, this *balance problem* may actually question the nature of divisive normalization in terms of other models. For instance, in Malo and Bertalmio (2018) we show that the divisive normalization may be seen as the stationary solution of *lower-level* Wilson-Cowan dynamics that do use a sensible unit-norm Gaussian interaction between units. This kind of questions are only raised, and solutions may be proposed, when testing interpretable models with model-related stimuli.

### 4.2. Using Naturalistic Databases Is Always a Problem?

Our criticism of naturalistic databases because their eventual imbalance and the problem in interpreting complicated stimuli in terms of models does not mean that we claim for an absolute rejection of these naturalistic databases. The case we studied here only suggests that one should not use the databases *blindly* as the only source of information, but in appropriate combination with well-selected artificial stimuli.

The use of carefully selected artificial stimuli may be considered as a safety-check of biological plausibility. Of course, our intention with the case studied here was not exhausting the search possibilities to claim that we obtained some sort of optimal solution. Instead, we just wanted to stress the fact that using the appropriate stimuli it is easy to propose modifications of the model that go in the right (biologically meaningful) direction, and still represent a competitive solution for the naturalistic database. This is an intuitive way to jump to other local minima which may be more biologically plausible in a very different region of the parameter space.

A sensible procedure would be alternating different learning epochs using natural and artificial data: while the large-scale naturalistic databases coming from the *image processing* community may enforce the main trends of the system, the specific small-scale artificial stimuli coming from the *vision science* community will fine-tune that first order approximation so that the resulting model has the appropriate features revealed by more specific experiments. In this context, standardization efforts such as those done by the CIE and the OSA organizations are really important to make this double-check. Examples include the data supporting the standard color observer (Smith and Guild, 1931; Stockman, 2017) and the standard spatial observer (Ahumada, 1996).

From a more general perspective, *image processing* applications do have a fundamental interest in *visual neuroscience* because these applications put into a broader context the relative relevance of the different phenomena described by classical psychophysics or physiology. For instance, one can check the variations in performance by testing vision models of different complexity, e.g., with or without this or that nonlinearity accounting for some specific perceptual effect/ability. This approach oriented to check different perceptual modules in specific applications has been applied in image quality databases (Watson and Malo, 2002), but also in other domains such as perceptual image and video compression (Malo et al., 2000a,b, 2001, 2006), or in perceptual image denoising and enhancement (Gutiérrez et al., 2006; Bertalmio, 2014). These different applications show the relative relevance of improvements in masking models with regard to better CSFs or including more sensible motion estimation models in front of better texture perception models.

### 4.3. Are All the Databases Created Equal?

The case analyzed in this work illustrates the effect of (naively) using a database where texture masking is probably under-represented. The lesson to learn is that one has to take into account the phenomena for which database was created, or, equivalently, the absence of specific phenomena to address.

With this in mind, one could imagine what kind of artificial stimuli are needed to improve the results. Or alternatively, which other naturalistic databases are required as complementary check since they are more focused on other kind of perceptual behavior.

Some examples to illustrate this point: databases with controlled observation distance or accurate chromatic calibration such as Pedersen (2015) are more appropriate to set the spatial frequency bandwidth of the models in achromatic and chromatic channels. Databases with spectrally controlled illumination pairs (Laparra et al., 2012; Gutmann et al., 2014; Laparra and Malo, 2015) are appropriate to address chromatic adaptation models. Databases with high-dynamic range (Korshunov et al., 2015; Cerda-Company et al., 2016) will be more appropriate to point out the need of the nonlinearity of brightness perception. Finally, databases where visibility of incremental patterns was carefully controlled in contrast terms (Alam et al., 2014) are the best option to fit masking models as opposed to generic subjectively-rated image distortion databases.

### 4.4. Final Remarks

Previous literature (Rust and Movshon, 2005) criticized the use of too complex natural stimuli in vision science experiments because the statistics of such stimuli are difficult to control and conclusions may be biased by the interaction between this poorly controlled input and the complexities of the neural model under consideration.

In line with such precautions on the use of natural stimuli, here we make a different point: the general criticism to blind use of machine learning in large-scale databases (related to the proper balance in the data) also applies when using subjectively rated image databases to fit vision models. Using a variety of natural scenarios and distortions cannot guarantee that specific behaviors are properly represented, thus remaining hidden in the vast amount of data. In such situation, models that seem to have the right structure may miss these basic phenomena. Instead of trying to explicitly include model-oriented artificial stimuli in the large database to fix the unbalance, it is easier to address the issue by using the model-oriented artificial stimuli in illustrative experiments specifically intended to test some parameters of the model.

The case study considered here suggests that artificial stimuli, motivated by specific phenomena or by features of the model, may help both to (a) stress the problems that remain in models fitted to imbalanced natural image databases, and (b) to suggest modifications in the models. Incidentally, this is also an argument in favor of interpretable parametric models as opposed to data-driven pure-regression models. A sensible procedure to fit general purpose vision models would be alternating different fitting strategies using (a) uncontrolled natural stimuli, but also (b) well-controlled artificial stimuli to check the biological plausibility at each point.

In conclusion, predicting subjective distances between images may be a trivial regression problem, but using these large-scale databases to fit plausible models may take more than that: for instance, a vision scientist in the loop doing the proper fine-tuning of interpretable models using the classical artificial stimuli.

## Author Contributions

JM conceived the work, prepared the data and code for the experiments, and contributed to the interpretation of the results and manuscript writing. MM-G ran the experiments. MB contributed to the manuscript writing and to the criticism of blind machine-learning-like approaches.

### Conflict of Interest Statement

The authors declare that the research was conducted in the absence of any commercial or financial relationships that could be construed as a potential conflict of interest.

## Footnotes

^1^A non exhaustive list of references and links to subjective quality databases includes (Webster et al., 2001; Ponomarenko et al., 2009, 2015; Larson and Chandler, 2010; Pedersen, 2015; Ghadiyaram and Bovik, 2016).

^2^For instance, analyzing images by choosing Gabors or different wavelets, and by choosing different ways to sample the retinal and the frequency spaces, definitely leads to different distributions of the energy over the subbands.

^3^It is true that **Model A** only included intra-band relations, but note also that, even though we wanted to introduce more general kernels in **Model B** for future developments, the solution to the qualitative problem considered here basically came from including 𝔻_***w***_ in *H* (not from sophisticated cross-subband weights). The other ingredients, ***b*** and ***c*** were already present in **Model A**.
