# Supplementary material for: In Praise of Artifice Reloaded: Caution With Natural Image Databases in Modeling Vision
Source: Front Neurosci. 2019 Feb 18;13:8. doi: 10.3389/fnins.2019.00008 (PMC6414813; doi:10.3389/fnins.2019.00008)
Supplement: Supplementary file 1 [file Data_Sheet_1.pdf]

Frontiers in Neuroscience

Research Article
11 February 2019

In Praise of Artifice Reloaded: Caution with Natural Image Databases in Modeling Vision (Supplemental Datasheet)

Marina Martinez-Garcia[1,2], **Marcelo Bertalmío**[3], **Jesús Malo**[1*]

[1] *Image Processing Lab., Universitat de València, Spain.*
[2] *Instituto de Neurociencias, CSIC, Spain.*
[3] *Dept. Inform. Comm. Technol., UPF, Barcelona, Spain.*

Correspondence*:
Jesus Malo
Image Processing Lab, Universitat de València, Catedrático Escardino, Paterna, València, 46980, Spain,
jesus.malo@uv.es

ABSTRACT

This is a Supplemental Datasheet with the explicit formulation of **Model A** and **Model B** considered in the main text. A Matlab implementation of both models including their derivatives and inverse is available at `http://isp.uv.es/docs/BioMultiLayer_L_NL_a_and_b.zip`. This code also includes numerical checks of the analytical results presented here.

1 FORMULATION OF MODELS

As stated in the *Material and Methods* section of the main text, **Model A** and **Model B** consist of a cascade of linear + nonlinear layers and these models only differ in the nonlinear part of the layer based on wavelet transform, which should account for the frequency and the orientation masking.

In this formulation we follow the two suggestions made in (**Martinez-Garcia et al.**, 2018): (a) we provide not only the forward transform, but also all the Jacobians and the inverse; and (b) we use matrix notation. We simply list the results for the Jacobian and the inverse with no proof, just for the reader convenience. However, in the provided toolbox, `BioMultiLayer_L_NL_a_and_b.zip`, there is a routine that numerically checks the Jacobian and compares the inverse to the actual input.

2 FORWARD TRANSFORM

Here, in each layer we use convolutional filters for the linear part, $\mathcal{L}$, and the canonical Divisive Normalization for the nonlinear part, $\mathcal{N}$. The forward transforms performed by each specific layer are described below:

Layer 1: Brightness from Radiance

$$\begin{aligned} \mathcal{L}^{(1)} \equiv \qquad \boldsymbol{y}^1 &= L^1 \cdot \boldsymbol{x}^0 \\ \mathcal{N}^{(1)} \equiv \qquad \boldsymbol{x}^1 &= K(\boldsymbol{y}^1) \cdot \mathbb{D}^{-1}_{\left(\boldsymbol{b}^1 + H^1 \cdot \boldsymbol{y}^{1^{\gamma^1}}\right)} \cdot \boldsymbol{y}^{1^{\gamma^1}} \end{aligned} \qquad (1)$$

where, L^1 is a matrix with the color matching functions for each spatial location. In particular, restricting ourselves to achromatic information, the only required color matching function would be the spectral sensitivity V_λ (**Wyszecki and Stiles**, 1982; **Fairchild**, 2013), leading to the luminance in each spatial location. The nonlinear part is the canonical Divisive Normalization, where the Hadamard products and quotients have been expressed using diagonal matrices: note that $\boldsymbol{a} \odot \boldsymbol{b} = \mathbb{D}_{\boldsymbol{a}} \cdot \boldsymbol{b} = \mathbb{D}_{\boldsymbol{b}} \cdot \boldsymbol{a}$ and $\mathbb{D}_{\boldsymbol{v}}$ is a diagonal matrix with the vector $\boldsymbol{v}$ in the diagonal (**Martinez-Garcia et al.**, 2018; **Minka**, 2001). The global scaling matrix $K(\boldsymbol{y}^1) = \kappa \left(\mathbb{D}_{\boldsymbol{b}^1} + \mathbb{D}_{\left(\frac{\beta}{d}\mathbb{1}\cdot\boldsymbol{y}^{1\gamma^1}\right)} + I \right)$, just ensures that the maximum brightness value (for normalized luminance equal to 1) is κ. The role of the interaction kernel in the denominator $H^1 = \left(\frac{\beta}{d}\mathbb{1} + I\right)$, where $\mathbb{1}$ is the all-ones $d \times d$ matrix, and I is the identity matrix, is setting the anchor for the brightness adaptation. With this kernel in the denominator the anchor luminance is related to the average luminance energy $\left(\boldsymbol{b}^1 + \frac{\beta}{d}\mathbb{1} \cdot \boldsymbol{y}^{1\gamma^1}\right)$. The effect of this nonlinear transform is a Weber-like adaptive saturation (**Abrams et al.**, 2007). Similar nonlinear behavior can be assumed for the opponent chromatic channels (**Fairchild**, 2013; **Stockman and Brainard**, 2010; **Laparra et al.**, 2012), but we didnt implemented the color version of the model.

Layer 2: Contrast from Brightness

$$\begin{aligned} \mathcal{L}^{(2)} \equiv \qquad \boldsymbol{y}^2 &= L^2 \cdot \boldsymbol{x}^1 \\ \mathcal{N}^{(2)} \equiv \qquad \boldsymbol{x}^2 &= \mathbb{D}^{-1}_{\left(\boldsymbol{b}^2 + H^2 \cdot \boldsymbol{y}^2\right)} \cdot \boldsymbol{y}^2 \end{aligned} \tag{2}$$

where the linear stage computes the deviation of point-wise brightness with regard to the local brightness through $L^2 = I - \mathcal{H}^n$, and this kernel in the *numerator*, $\mathcal{H}^n$, represents the convolution by a two-dimensional Gaussian. The normalization through $H^2 = \mathcal{H}^d \cdot (I - \mathcal{H}^n)^{-1}$, where the kernel in the *denominator*, $\mathcal{H}^d$, is another two-dimensional Gaussian kernel, leads to the standard definition of contrast: normalization of the deviation of brightness by the local brightness.

Layer 3: Contrast sensitivity and spatial masking

$$\begin{aligned} \mathcal{L}^{(3)} \equiv \qquad \boldsymbol{y}^3 &= L^3 \cdot \boldsymbol{x}^2 \\ \mathcal{N}^{(3)} \equiv \qquad \boldsymbol{x}^3 &= \mathbb{D}_{\text{sign}(\boldsymbol{y}^3)} \cdot \mathbb{D}^{-1}_{\left(\boldsymbol{b}^3 + H^3 \cdot |\boldsymbol{y}^3|^{\gamma^3}\right)} \cdot |\boldsymbol{y}^3|^{\gamma^3} \end{aligned} \tag{3}$$

where L^3 is the convolution matrix equivalent to the application of a Contrast Sensitivity Function (CSF) (**Campbell and Robson**, 1968). The rows of this matrix consist of displaced versions of center-surround (LGN-like) receptive fields (impulse response of the CSF **Martinez-Uriegas** (1997)). The kernel in the denominator, H^3, represents the convolution by another two-dimensional Gaussian that computes the local contrast energy that masks the responses in high-energy environments.

Layer 4: Wavelet analysis and frequency masking

$$\begin{aligned} \mathcal{L}^{(4)} \equiv \qquad \boldsymbol{y}^4 &= L^4 \cdot \boldsymbol{x}^3 \\ \mathcal{N}^{(4)} \equiv \qquad \boldsymbol{x}^4 &= \mathbb{D}_{\text{sign}(\boldsymbol{y}^4)} \cdot \mathbb{D}^{-1}_{\left(\boldsymbol{b}^4 + H^4 \cdot |\boldsymbol{y}^4|^{\gamma^4}\right)} \cdot |\boldsymbol{y}^4|^{\gamma^4} \end{aligned} \tag{4}$$

where L^4 is the matrix of Gabor-like receptive fields corresponding to V1-like sensors (**Simoncelli and Adelson**, 1990). The kernel in the denominator, H^4, represents the masking interaction between sensors

tuned to different space, frequency and orientation (**Watson and Solomon**, 1997). The focus of this paper is in the effect of training this 4th stage in large-scale naturalistic databases.

In **Model A** the kernel of the Divisive Normalization is restricted to intra-band submatrices. **Model B** consists of (a) adding a global scaling factor (diagonal matrix) to set the dynamic range of the output, and (b) generalizing the interaction kernel,

$$\begin{aligned} \mathcal{N}_B(\boldsymbol{e}) = K(\boldsymbol{e}^\star)\cdot\mathcal{N}_A(\boldsymbol{e}) &= K(\boldsymbol{e}^\star)\cdot\mathbb{D}_{\text{sign}(\boldsymbol{y})}\cdot\mathbb{D}^{-1}_{(\boldsymbol{b}+H_G\cdot\boldsymbol{e})}\cdot\boldsymbol{e} \\ where & \\ K(\boldsymbol{e}^\star) &= \mathbb{D}_{\boldsymbol{\kappa}}\cdot\mathbb{D}_{(\boldsymbol{b}+H_G\cdot\boldsymbol{e}^\star)}\cdot\mathbb{D}^{-1}_{\boldsymbol{e}^\star} \\ H_G &= \mathbb{D}_{\boldsymbol{c}}\cdot\left[H_{\boldsymbol{p}}\odot H_f\odot H_\phi\odot C_{\text{int}}\right]\cdot\mathbb{D}_{\boldsymbol{w}} \end{aligned} \tag{5}$$

where the *global scaling vector*, $\boldsymbol{\kappa}$ (that determines the dynamic range of the output) is obtained from the intra-subband average of the response to natural images, $|\boldsymbol{x}|$, in **Model A**, and the *reference vector*, $\boldsymbol{e}^\star$ (that describes the dynamic range of the input to $\mathcal{N}_B$) may be independent of the input, e.g., a global normalization constant vector, or, it may depend on each specific input. In the first case the fixed normalization may be obtained from the intra-subband average of natural images in $|\boldsymbol{y}|^\gamma$. In the second case, similarly to Layer 1, dependence with the input can be seen as auto-normalization as opposed to fixed normalization. In the auto-normalization case, the dynamic range of the input may be set from the average value in each subband (as in Layer 1, where the anchor luminance depends on the average luminance). The averages over the subbands can be computed as $\boldsymbol{e}^\star = \mathbb{D}_{\frac{1}{d_w}}\cdot\mathbb{1}_w\cdot\boldsymbol{e}$. Where $\mathbb{1}_w$ is a block-diagonal matrix with *all-ones* in the diagonal blocks corresponding to each subband and $\mathbb{D}_{\frac{1}{d_w}}$ is a diagonal matrix that divides the corresponding sum by the dimension of the wavelet subband, thus leading to the average. This apparently complicated matrix expression for the average is just to simplify the derivative with regard to the stimulus, which reduces to the constant matrix $\mathbb{D}_{\frac{1}{d_w}}\cdot\mathbb{1}_w$.

The generalized interaction kernel is given by the modulation (Hadamard, element-wise product) of three Gaussian kernels over the locations, $\boldsymbol{p}$, scales f, and orientations ϕ of the wavelet-like coefficients. These Gaussian kernels have normalization constants so that they have unit volume in their definition domain. In the case of spatial kernels, as the number of sensors per subband depends on the subband, the amplitude of the Gaussian kernel also depends on the subband. Given the already nonlinear nature of the input $\boldsymbol{e}$, this difference in the normalization factor may mean that some subbands are over-weighted with regard to others. That is why we included other matrices (the full matrix C_{int} and the diagonal matrix $\mathbb{D}_{\boldsymbol{w}}$) to compensate these effects if necessary. Note that $\mathbb{D}_{\boldsymbol{w}}$ applies column-wise weights on the final kernel, or equivalently, it selectively weights the energy of the subbands in the input vector $\boldsymbol{e}$. This means that it can be used to moderate the effect of the a specific subband if it is too big. More importantly, one could act on a specific block of the full matrix, C_{int}, if the relation between two specific subbands should be modified. We did not have to do that in this work for a qualitative fix of the behavior, i.e., C_{int} remained an all-ones matrix. Finally, the global normalization of each row is controlled by the vector in the diagonal matrix $\mathbb{D}_{\boldsymbol{c}}$.

3 JACOBIAN MATRIX WITH REGARD TO THE STIMULUS

In feed-forward networks the Jacobian $\nabla_{\boldsymbol{x}^0}S(\boldsymbol{x}^0)$ reduces to the knowledge of the Jacobian of each stage, $\nabla_{\boldsymbol{y}^i}\mathcal{N}^{(i)}(\boldsymbol{y}^i)$ (**Martinez-Garcia et al.**, 2018). In this work in the modified **Model B**, the only term yet to be specified after the results in (**Martinez-Garcia et al.**, 2018) is, $\nabla_{\boldsymbol{y}}\mathcal{N}_B(\boldsymbol{y})$. If we call

$\boldsymbol{x}_A = \mathcal{N}_A(\boldsymbol{y}, H_G) = \mathbb{D}_{\text{sign}(\boldsymbol{y})} \cdot \mathbb{D}^{-1}_{(\boldsymbol{b}+H_G\cdot\boldsymbol{e})} \cdot \boldsymbol{e}$, we have:

$$\nabla_{\boldsymbol{y}} \mathcal{N}_B \;=\; K(\boldsymbol{e}^\star) \cdot \nabla_{\boldsymbol{y}} \mathcal{N}_A(\boldsymbol{y}) + \mathbb{D}_{\boldsymbol{x}_A} \cdot \nabla_{\boldsymbol{y}} K(\boldsymbol{e}^\star) \tag{6}$$

$$where$$

$$\begin{aligned}
\nabla_{\boldsymbol{y}} \mathcal{N}_A(\boldsymbol{y}) &= \mathbb{D}_{\text{sign}(\boldsymbol{y})} \cdot \mathbb{D}^{-1}_{(\boldsymbol{b}+H_G\cdot\boldsymbol{e})} \cdot \left[I - \mathbb{D}_{\left(\frac{\boldsymbol{e}}{\boldsymbol{b}+H_G\cdot\boldsymbol{e}}\right)} \cdot H_G \right] \cdot \mathbb{D}_{\gamma|\boldsymbol{y}|^{\gamma-1}} \cdot \mathbb{D}_{\text{sign}(\boldsymbol{y})} \\
\nabla_{\boldsymbol{y}} K(\boldsymbol{e}^\star) &= \nabla_{\boldsymbol{e}} K(\boldsymbol{e}^\star) \cdot \mathbb{D}_{\gamma|\boldsymbol{y}|^{\gamma-1}} \cdot \mathbb{D}_{\text{sign}(\boldsymbol{y})} \\
\nabla_{\boldsymbol{e}} K(\boldsymbol{e}^\star) &= \begin{cases} 0 & \text{in case } \boldsymbol{e}^\star \text{ is constant} \\ \mathbb{D}_{\boldsymbol{\kappa}} \cdot \left(\mathbb{D}^{-1}_{\boldsymbol{e}^\star} \cdot H_G - \mathbb{D}_{(\boldsymbol{b}+H_G\cdot\boldsymbol{e}^\star)} \cdot \mathbb{D}^{-2}_{\boldsymbol{e}^\star} \right) \cdot \mathbb{D}_{\frac{1}{dw}} \cdot \mathbb{1}_w & \text{for auto-normalization} \end{cases}
\end{aligned}$$

4 JACOBIAN MATRICES WITH REGARD TO THE PARAMETERS

The set of parameters of the modified nonlinearity of the 4-th layer is $\theta = \{\boldsymbol{\kappa}, \boldsymbol{b}, \gamma, \boldsymbol{\sigma_p}, \boldsymbol{\sigma}_f, \boldsymbol{\sigma}_\phi, \boldsymbol{c}, \boldsymbol{w}\}$. In this set, $\boldsymbol{\kappa}$ is vector of weights that determines the scale of the different subbands of the response. The vector, $\boldsymbol{b}$, is the set of semisaturations (one per sensor). The exponent γ determines the strength of the excitation/inhibition. The vector $\boldsymbol{\sigma_p}$ contains the widths of the spatial interaction kernels in $H_{\boldsymbol{p}}$ affecting each sensor. Similarly, the vector $\boldsymbol{\sigma_f}$ contains the widths of the frequency interaction kernels in H_f, and the vector $\boldsymbol{\sigma_\phi}$ contains the widths of the angular interaction kernels in H_ϕ. The vector $\boldsymbol{c}$ describes the amplitudes of the interaction kernels for the sensors (that goes in the diagonal matrix $\mathbb{D}_{\boldsymbol{c}}$) in Eq. 6 in the main text. And finally, the vector $\boldsymbol{w}$ determines the specific weight of each linear coefficient in the masking.

The list of Jacobian matrices is the following:

$$\nabla_{\boldsymbol{\kappa}} \mathcal{N}_B = \mathbb{D}_{\left(sign(\boldsymbol{y}) \odot \frac{\boldsymbol{b}+H_G\cdot\boldsymbol{e}^\star}{\boldsymbol{e}^\star} \odot \frac{\boldsymbol{e}}{\boldsymbol{b}+H_G\cdot\boldsymbol{e}}\right)} \tag{7}$$

$$\nabla_{\boldsymbol{b}} \mathcal{N}_B = \mathbb{D}_{sign(\boldsymbol{y})} \cdot \kappa \cdot \mathbb{D}^{-1}_{\boldsymbol{e}^\star} \cdot \mathbb{D}_{\boldsymbol{e}} \cdot \left[\mathbb{D}^{-1}_{(\boldsymbol{b}+H_G\cdot\boldsymbol{e})} - \mathbb{D}_{(\boldsymbol{b}+H_G\cdot\boldsymbol{e}^\star)} \cdot \mathbb{D}^{-2}_{(\boldsymbol{b}+H_G\cdot\boldsymbol{e})} \right] \tag{8}$$

$$\nabla_\gamma \mathcal{N}_B = \begin{cases} \mathbb{D}_{sign(\boldsymbol{y})} \cdot K(\boldsymbol{e}^\star) \cdot \mathbb{D}^{-1}_{\mathcal{D}(\boldsymbol{e})} \cdot \left[\mathbb{D}_{log(|\boldsymbol{y}|)} - \mathbb{D}^{-1}_{\mathcal{D}(\boldsymbol{e})} \cdot \mathbb{D}_{H_G\cdot\mathbb{D}_{\boldsymbol{e}}\cdot log(|\boldsymbol{y}|)} \right] \cdot \boldsymbol{e} \\ \text{in case } \boldsymbol{e}^\star \text{ is constant} \\ \\ \mathbb{D}_{sign(\boldsymbol{y})} \cdot (A+B) \cdot \boldsymbol{e} \\ \text{in case of auto-normalization, where:} \\ \quad A = K(\boldsymbol{e}^\star) \cdot \mathbb{D}^{-1}_{\mathcal{D}(\boldsymbol{e})} \cdot \left[\mathbb{D}_{log(|\boldsymbol{y}|)} - \mathbb{D}^{-1}_{\mathcal{D}(\boldsymbol{e})} \cdot \mathbb{D}_{H_G\cdot\mathbb{D}_{\boldsymbol{e}}\cdot log(|\boldsymbol{y}|)} \right] \\ \quad B = \mathbb{D}_\kappa \cdot \mathbb{D}^{-1}_{\boldsymbol{e}^\star} \cdot \left[\mathbb{D}_{\left(H_G\cdot\mathbb{D}_{\frac{1}{dw}}\cdot\mathbb{1}_w\cdot\mathbb{D}_{\boldsymbol{e}}\cdot log(|\boldsymbol{y}|)\right)} - \mathbb{D}_{\mathcal{D}(\boldsymbol{e}^\star)} \cdot \mathbb{D}^{-1}_{\boldsymbol{e}^\star} \cdot \mathbb{D}_{\left(\mathbb{D}_{\frac{1}{dw}}\cdot\mathbb{1}_w\cdot\mathbb{D}_{\boldsymbol{e}}\cdot log(|\boldsymbol{y}|)\right)} \right] \cdot \mathbb{D}^{-1}_{\mathcal{D}(\boldsymbol{e})} \end{cases} \tag{9}$$

where $\mathcal{D}(\boldsymbol{e})$ stands for the denominator $\boldsymbol{b} + H_G \cdot \boldsymbol{e}$.

In the derivatives with regard to the parameters that control the generalized kernel H_G there are two separate cases: (a) parameters that model the rows of the kernel, and (b) parameters that model the columns of the kernel. The first case includes the widths of the Gaussian kernels in the rows of $H_{\boldsymbol{p}}$, H_f and H_ϕ; and the amplitude of each row (the scaling matrix $\mathbb{D}_{\boldsymbol{c}}$). The second case refers to the weights in the matrix $\mathbb{D}_{\boldsymbol{w}}$ that affect each column of the kernel.

In the first case, the Jacobian with regard to parameters of the rows is:

$$\nabla_{\boldsymbol{\theta}} \mathcal{N}_B = \mathbb{D}_{\left(\text{sign}(\boldsymbol{y})\odot\frac{\boldsymbol{e}}{\mathcal{D}(\boldsymbol{e})}\right)} \cdot \left(\mathbb{D}_{\left(\frac{\boldsymbol{\kappa}}{\boldsymbol{e}^\star}\right)} \cdot \text{diag}\left[\begin{pmatrix} \boldsymbol{e}^{\star\top} \\ \vdots \\ \boldsymbol{e}^{\star\top} \end{pmatrix} \cdot \left(\frac{\partial H_G}{\partial \boldsymbol{\theta}}\right)^\top \right] - \mathbb{D}^{-1}_{\mathcal{D}(\boldsymbol{e})} \cdot K(\boldsymbol{e}^\star) \cdot \text{diag}\left[\begin{pmatrix} \boldsymbol{e}^{\top} \\ \vdots \\ \boldsymbol{e}^{\top} \end{pmatrix} \cdot \left(\frac{\partial H_G}{\partial \boldsymbol{\theta}}\right)^\top \right] \right) \tag{10}$$

where, for each specific parameter, $\{\boldsymbol{\sigma_p}, \boldsymbol{\sigma}_f, \boldsymbol{\sigma}_\phi, \boldsymbol{c}\}$, we have:

$$\frac{\partial H_G}{\partial \boldsymbol{\sigma_p}} = \mathbb{D}_{\boldsymbol{c}} \cdot \left(H_f \odot H_\phi \odot C_{\text{int}} \odot \frac{\partial H_{\boldsymbol{p}}}{\partial \boldsymbol{\sigma_p}} \right) \cdot \mathbb{D}_{\boldsymbol{w}} \quad \text{where} \quad \left(\frac{\partial H_{\boldsymbol{p}}}{\partial \boldsymbol{\sigma_p}}\right)_{ij} = \frac{dp_1 dp_2}{\sigma_{\boldsymbol{p}_i}^{\;5}\, n_s\, 2\pi} \left(\Delta^2_{ij} - 2\,\sigma_{\boldsymbol{p}_i}^{\;2}\right) e^{-\frac{\Delta^2_{ij}}{2\,\sigma_{\boldsymbol{p}_i}^2}}$$

where $dp_1 dp_2$ is the area (in deg^2) of the discrete sampling cell for the sensors of the same scale as the i-th sensor; n_s is the number of subbands in the wavelet pyramid; and the departure Δ^2_{ij} is the 2-norm of the spatial distance between the i-th and the j-th sensors, $|\boldsymbol{p}_i - \boldsymbol{p}_j|^2$.

$$\frac{\partial H_G}{\partial \boldsymbol{\sigma}_f} = \mathbb{D}_{\boldsymbol{c}} \cdot \left(H_{\boldsymbol{p}} \odot H_\phi \odot C_{\text{int}} \odot \frac{\partial H_f}{\partial \boldsymbol{\sigma}_f} \right) \cdot \mathbb{D}_{\boldsymbol{w}} \quad \text{where} \quad \left(\frac{\partial H_f}{\partial \boldsymbol{\sigma}_f}\right)_{ij} = \frac{1}{\sigma_{f_i}^{\;4}\sqrt{2\pi}} \left(\Delta f^2_{ij} - \sigma_{f_i}^{\;2}\right) e^{-\frac{\Delta f^2_{ij}}{2\,\sigma_{f_i}^2}}$$

where the departure in frequency between the i-th and j-th sensors, Δf_{ij}, is measured in octaves.

$$\frac{\partial H_G}{\partial \boldsymbol{\sigma}_\phi} = \mathbb{D}_{\boldsymbol{c}} \cdot \left(H_{\boldsymbol{p}} \odot H_f \odot C_{\text{int}} \odot \frac{\partial H_\phi}{\partial \boldsymbol{\sigma}_\phi} \right) \cdot \mathbb{D}_{\boldsymbol{w}} \quad \text{where} \quad \left(\frac{\partial H_\phi}{\partial \boldsymbol{\sigma}_\phi}\right)_{ij} = \frac{d\phi}{\sigma_{\phi_i}^{\;4}\sqrt{2\pi}} \left(\Delta \phi^2_{ij} - \sigma_{\phi_i}^{\;2}\right) e^{-\frac{\Delta \phi^2_{ij}}{2\,\sigma_{\phi_i}^2}}$$

where $d\phi$ is the angular separation between different orientations in the wavelet pyramid and it is measured in the same units as the angular departure between sensors, $\Delta\phi_{ij}$.

$$\frac{\partial H_G}{\partial \boldsymbol{c}} = \left(H_{\boldsymbol{p}} \odot H_f \odot H_\phi \odot C_{\text{int}} \right) \cdot \mathbb{D}_{\boldsymbol{w}}$$

In the second case, the Jacobian with regard to parameters of the columns is:

$$\nabla_{\boldsymbol{w}} \mathcal{N}_B = \mathbb{D}_{\left(\text{sign}(\boldsymbol{y})\odot\frac{\boldsymbol{e}}{\mathcal{D}(\boldsymbol{e})}\right)} \cdot \left(\mathbb{D}_{\left(\frac{\boldsymbol{\kappa}}{\boldsymbol{e}^\star}\right)} \cdot \left(\frac{\partial H_G}{\partial \boldsymbol{w}}\right) \cdot \mathbb{D}_{\boldsymbol{e}} - \mathbb{D}^{-1}_{\mathcal{D}(\boldsymbol{e})} \cdot K(\boldsymbol{e}^\star) \cdot \left(\frac{\partial H_G}{\partial \boldsymbol{w}}\right) \cdot \mathbb{D}_{\boldsymbol{e}^\star} \right) \tag{11}$$

where, $\frac{\partial H_G}{\partial \boldsymbol{w}} = \mathbb{D}_{\boldsymbol{c}} \cdot \left(H_{\boldsymbol{p}} \odot H_f \odot H_\phi \odot C_{\text{int}} \right)$.

5 INVERSE

If the reference vector, $\boldsymbol{e}^\star$, is constant, the inverse has closed form:

$$\boldsymbol{y} = \mathcal{N}_B^{-1}(\,K(\boldsymbol{e}^\star)^{-1} \cdot |\boldsymbol{x}|\,) = \mathbb{D}_{\text{sign}(\boldsymbol{x})} \cdot \left[\left(I - \mathbb{D}_{(K(\boldsymbol{e}^\star)^{-1}\cdot|\boldsymbol{x}|)} \cdot H_G \right)^{-1} \cdot \mathbb{D}_{\boldsymbol{b}} \cdot K(\boldsymbol{e}^\star)^{-1} \cdot |\boldsymbol{x}| \right]^{\frac{1}{\gamma}} \tag{12}$$

On the contrary, in the case of auto-adaptation, when coming back from certain $\boldsymbol{x}$, the reference $\boldsymbol{e}^\star$ is unknown. Nevertheless, the inverse can still be obtained iteratively. Starting from certain guess $\boldsymbol{e}^\star_0$ (e.g.,

the one for natural images), one can obtain a first guess for the inverse $\boldsymbol{y}_1$ using Eq. 12. From the n-th guess for the inverse, one can derive a new guess for the reference: $\boldsymbol{e}^{\star}_n = \mathbb{D}_{\frac{1}{d_w}} \cdot \mathbb{1}_w \cdot |\boldsymbol{y}_n|^{\gamma}$, and keep the iteration:

$$\boldsymbol{y}_{n+1} = \mathcal{N}_B^{-1}(K(\boldsymbol{e}^{\star}_n)^{-1} \cdot \boldsymbol{x}) \qquad (13)$$

where $\mathcal{N}_B^{-1}$ is computed using Eq. 12.

REFERENCES

Abrams, A. B., Hillis, J. M., and Brainard, D. H. (2007), The relation between color discrimination and color constancy: When is optimal adaptation task dependent?, *Neural Computation*, 19, 10, 2610–2637

Campbell, F. and Robson, J. (1968), Application of Fourier analysis to the visibility of gratings, *Journal of Physiology*, 197, 551–566

Fairchild, M. (2013), Color Appearance Models, The Wiley-IS&T Series in Imaging Science and Technology (Wiley)

Laparra, V., Jiménez, S., Camps-Valls, G., and Malo, J. (2012), Nonlinearities and adaptation of color vision from sequential principal curves analysis, *Neural Computation*, 24, 10, 2751–2788

Martinez-Garcia, M., Cyriac, P., Batard, T., Bertalmio, M., and Malo, J. (2018), Derivatives and inverse of cascaded linear+nonlinear neural models, *PLoS ONE. 13(10): e0201326. https://doi.org/10.1371/journal.pone.0201326*

Martinez-Uriegas, E. (1997), Color detection and color contrast discrimination thresholds, *Proc. OSA Meeting*, 81

Minka, T. P. (2001), Old and new matrix algebra useful for statistics, Technical report

Simoncelli, E. and Adelson, E. (1990), Subband Image Coding (Kluwer Academic Publishers, Norwell, MA), chapter Subband Transforms, 143–192

Stockman, A. and Brainard, D. (2010), OSA Handbook of Optics (3rd. Ed.) (McGraw-Hill, NY), chapter Color vision mechanisms, 147–152

Watson, A. and Solomon, J. (1997), A model of visual contrast gain control and pattern masking, *JOSA A*, 14, 2379–2391

Wyszecki, G. and Stiles, W. (1982), Color Science: Concepts and Methods, Quantitative Data and Formulae (John Wiley & Sons, New York)
